# Interaction of the La-related protein Slf1 with colliding ribosomes maintains translation of oxidative-stress responsive mRNAs

**DOI:** 10.1093/nar/gkad272

**Published:** 2023-04-18

**Authors:** Martin D Jennings, Priya Srivastava, Christopher J Kershaw, David Talavera, Christopher M Grant, Graham D Pavitt

**Affiliations:** Division of Molecular and Cellular Function, School of Biological Sciences, The University of Manchester, Manchester, M13 9PT, UK; Division of Molecular and Cellular Function, School of Biological Sciences, The University of Manchester, Manchester, M13 9PT, UK; Division of Molecular and Cellular Function, School of Biological Sciences, The University of Manchester, Manchester, M13 9PT, UK; Division of Cardiovascular Sciences, School of Medical Sciences, The University of Manchester, Manchester, M13 9PT, UK; Division of Molecular and Cellular Function, School of Biological Sciences, The University of Manchester, Manchester, M13 9PT, UK; Division of Molecular and Cellular Function, School of Biological Sciences, The University of Manchester, Manchester, M13 9PT, UK

## Abstract

In response to oxidative stress cells reprogram gene expression to enhance levels of antioxidant enzymes and promote survival. In *Saccharomyces cerevisiae* the polysome-interacting La-related proteins (LARPs) Slf1 and Sro9 aid adaptation of protein synthesis during stress by undetermined means. To gain insight in their mechanisms of action in stress responses, we determined LARP mRNA binding positions in stressed and unstressed cells. Both proteins bind within coding regions of stress-regulated antioxidant enzyme and other highly translated mRNAs in both optimal and stressed conditions. LARP interaction sites are framed and enriched with ribosome footprints suggesting ribosome–LARP–mRNA complexes are identified. Although stress-induced translation of antioxidant enzyme mRNAs is attenuated in *slf1*Δ, these mRNAs remain on polysomes. Focusing further on Slf1, we find it binds to both monosomes and disomes following RNase treatment. *slf1*Δ reduces disome enrichment during stress and alters programmed ribosome frameshifting rates. We propose that Slf1 is a ribosome-associated translational modulator that stabilises stalled/collided ribosomes, prevents ribosome frameshifting and so promotes translation of a set of highly-translated mRNAs that together facilitate cell survival and adaptation to stress.

## Graphical Abstract

**Graphical Abstract:**
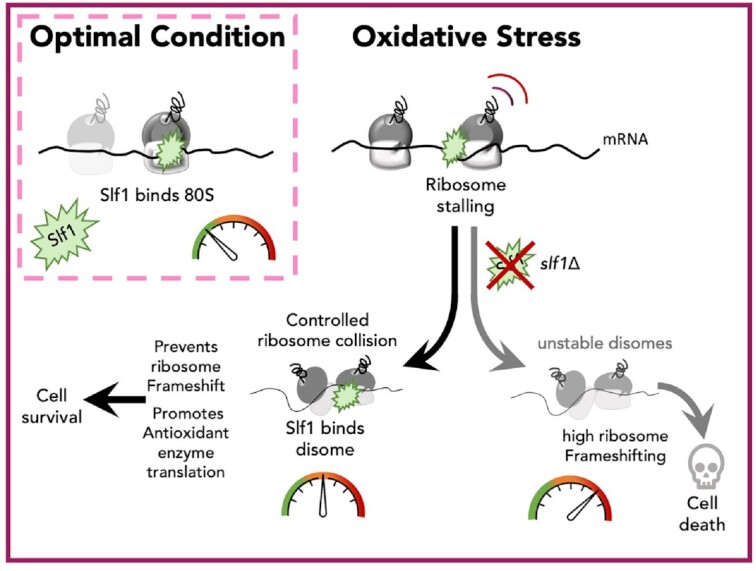
LaRP Slf1 stabilises ribosomes translating antioxidant enzyme mRNAs to promote cellular adaptation to stress.

## INTRODUCTION

Cells sense and respond rapidly to changes in their internal and external environments. Reactive oxygen species can be generated internally by aerobic respiration, or by external agents, such as hydrogen peroxide (hereafter peroxide). As oxidative damage to cellular components can cause cell death, organisms have evolved adaptive responses to oxidative stress, which include reprogramming both transcription and translation (1,2). *Saccharomyces cerevisiae* is an important model system to study these responses (2). Transcriptional control in response to peroxide is well-described and mediated largely by coordinated upregulation of antioxidant mRNAs that are targets of the transcriptional activator Yap1, accompanied by down-regulation of ribosomal protein mRNAs that reduce ribosome synthesis (3). At the same time there is a global reduction in protein synthesis. In common with mammalian cell responses to stress, yeast cells exposed to peroxide regulate both protein synthesis initiation and elongation. Initiation control acts via enhanced phosphorylation of the translation factor eIF2 (1). This is mediated by the sole eIF2 kinase Gcn2 and reduces rates of translation initiation globally while enabling some translation to continue as part of an integrated stress response (ISR), described below. Translation elongation is slowed in multiple ways which leads to increased ribosome stalls and collisions. For example, ribosome profiling has shown that ribosomes stall preferentially at proline codons in response to peroxide (4). In addition elongation factor eEF2 is phosphorylated during oxidative stress (4), which together correlate with earlier ribosome transit time measurements showing translation elongation slows even in the absence of Gcn2 (1). Additionally, a fraction of ribosomal proteins are ubiquitinated at multiple modification sites during stress which may further contribute to the observed slowed elongation rates (5,6). Finally, elongation inhibition may be directly coupled to initiation control as the Gcn2 activator protein Gcn1 was found to bind to stalled/collided ribosomes, called disomes (7). Reducing initiation rates when elongation is stalled should limit further accumulation of stalled/collided ribosomes which may otherwise activate ribosome associated quality control (RQC) pathways that can lead to mRNA decay and promote removal of stalled ribosomes (8).

The repressive mechanisms described above do not explain how antioxidant protein mRNAs, that are transcriptionally enhanced during stress overcome global inhibition of translation. One translational control mechanism is via altered translation of short upstream open reading frames (uORFs) that promote translation of regulated mRNAs during times of stress. *GCN4* in yeast and mammalian *ATF4* exemplify this mechanism, which is known as general amino acid control, and the ISR in yeast and mammals respectively (9,10). However many yeast mRNAs that are translationally active during peroxide stress lack uORFs (11), implying that other mechanisms operate to ensure these mRNAs are translated. Even where uORFs are present to enhance ribosome recruitment, this does not explain how translation elongation blocks are overcome. It was shown that deletion of either one of the yeast La-related proteins (LARPs) Slf1 or Sro9 confers enhanced cell sensitivity to peroxide, with loss of Slf1 conferring acute sensitivity. Further experiments, summarised below, implicate Slf1 as a positive regulator of the oxidative stress translational control response that promotes translation of stress response mRNAs (12).

LARPs are a family of RNA-binding proteins implicated in both translational control and disease (13,14). Each bears a La motif (LaM) necessary for RNA-binding and most contain additional domains not shared with the yeast counterparts. Human LARP1 confers negative regulation of mRNA translation, via binding of its DM15 domain to a class of mRNAs that possess a common 5′ terminal oligopyrimidine sequence (5′TOP) (15). 5′TOP mRNAs include translation factors and ribosomal proteins, and the LARP1 DM15 binds to down-regulate expression of these mRNAs during stress (16,17). In addition, LARP1 has been found to bind within the ORFs of other mRNAs (18). LARP1 has been associated with repressed mRNAs (19), as well as promoting translation of targets (20), and it is dysregulated during cancer (21). These findings suggest that LARP1 is a multifunctional protein with both activating and repressing roles that are not yet fully explained (22). The simpler domain structure of the yeast LARPs provides an opportunity to unravel a core role of the LaM. Both yeast LARPs, Slf1 and Sro9, are broadly homologous to each other over their entire lengths and, in addition to their shared LaM, possess amino-terminal regions implicated in ribosome binding (Supplementary Figure S1A). Both proteins are polysome-bound and remain so during acute oxidative stress (23,24). Polysome profiles of *slf1Δ* cells showed acute sensitivity to peroxide as ribosome run-off was enhanced (24). RNA immuno precipitation and microarrays (25) or RNA-sequencing (RIP-seq) (24) identified that Slf1 target RNAs include the transcriptionally induced antioxidant enzymes, while complementary whole-cell proteomics mass spectrometry showed that the stress-induced translation of antioxidant enzymes was significantly attenuated in *slf1Δ* cells (24). Together these results suggested an active role for Slf1 binding to ribosomes and promoting the translation of mRNAs needed for the normal cellular response to oxidative stress, but did not provide clues to the mechanism.

Here, we determined the RNA-binding patterns of the yeast LARPs by performing PAR-CLIP for Slf1 and Sro9 in exponentially growing cells and during the initial acute phase of oxidative stress when translation initiation is attenuated. We find that both factors bind within ORFs of an overlapping set of actively translated mRNAs including stress-response mRNAs transcriptionally induced by stress. The binding sites are typically framed similar to ribosome footprints and are enriched towards ORF 3′ ends. Meta-analyses of both ribosome 80S and disome footprints indicates that the LARP-bound sites are enriched with a significant fraction of both ribosomes and disomes. Although *slf1Δ* cells fail to induce antioxidant protein levels, ribosome engagement with target mRNAs is maintained, suggesting ribosomes initiate but may stall and then fail to complete protein synthesis. Ribosome-associated quality control (RQC) is responsible for recognizing, disassembling, and recycling stalled/collided ribosomes (8,26). The RQC factor Hel2 is enriched on the yeast LARP-targeted mRNAs with a similar enrichment towards ORF 3′ ends. We found that Slf1 is enriched on disomes and that the increase in disome abundance following stress is altered in an *slf1*Δ strain similarly to that seen for cells lacking RQC factors Hel2 or Mbf1. As Mbf1 acts to prevent ribosome frameshifting at stalling sequences, we assessed whether Slf1 modulates programmed ribosome frameshifting and found that it does help maintain ribosomes in the 0 frame. Together these data suggest a model whereby the LARPs interact with a sub-set of ribosomes on highly translated mRNAs and that Slf1 can enhance disome stability and ensure stalled ribosomes maintain their correct reading frame. Together these actions promote the synthesis of proteins, including antioxidant enzymes, needed for a timely response to oxidative stress.

## MATERIALS AND METHODS

### Biological resources

*Yeast strains*: Strain genotypes and sources are listed in Supplementary Table S7. All strains are derived from BY4741 (*MAT***a**). TAP-tagged strains used for PAR-CLIP (GP5991, GP6482 and GP6797) bear C-terminal tags integrated into the genome at the natural locus under their own promoters, as used previously (12,24). Except where indicated deletions used were KanMX4 gene replacements of the entire ORF.

CRISPR/Cas9 mediated deletion of the *SLF1* ORF was achieved by co-transformation pAV2713 (Supplementary Table S8) and the deletion repair oligo (Supplementary Table S9). Transformants were selected on SC–leucine medium. Deletions were confirmed by genomic extraction and PCR with *SLF1* up and down oligos (Supplementary Table S8) followed by Sanger sequencing. Other strains were made by transformation using plasmids (Supplementary Table S8) following standard transformation procedures.

*Plasmid construct generation*: Cas9 gRNA plasmid pAV2713 was generated as per Anand et al (27). Oligonucleotides (Supplementary Table S9) were designed to a Cas9 PAM site using the ATUM tool (https://www.atum.bio/eCommerce/cas9/input) with the setting Cas9 WT, NGG PAM. GTTTT was added to 3′ end of the forward oligo and GATCA to 3′ end of the reverse oligo Slf1_CRISPR_F + Slf1_CRISPR_R respectively (Supplementary Table S9). Oligos were combined at 25 μM and annealed by heating to 95°C for 5 min in 20 μl (1× ligase buffer in 10 mM Tris 1 mM EDTA) then cooling to room temperature. The annealed duplex was then ligated into the BplI site of bRA90 (27) (Supplementary Table S8). Correct insertion was confirmed by PCR using the CRISPR forward oligo with an AmpR primer (Supplementary Table S9).

### PAR-CLIP sample preparation

Duplicate samples of strains GP5991, GP6482 and GP6797 (Supplementary Table S7) were grown in 2 l SC-U-H + 2% glucose + 100 μM 4-thiouracil (4TU) to OD 0.3. An additional 900 μM 4TU was then added and cells were grown for a further 2 hours. During the final 15 minutes, treated samples were stressed with addition of 0.4 mM H_2_O_2_. Cells were pelleted by centrifugation and resuspended in 50 ml ice-cold SC + 3% glucose + double concentration amino acid supplement. Cells were spread out at a depth of 1 cm in a culture dish and cross-linked on ice at 365 nM with 12 J/cm^2^. Cells were then pelleted again, frozen and ground under liquid Nitrogen in a cryogenic mill. Ground cells were resuspended in IP 1× buffer (30 mM HEPES pH7.5, 100 mM KCl, 10% glycerol + protease inhibitors) and clarified at 20 000 × g. RNA and DNA was initially trimmed by addition of 40 U of DNase and RNase T1 to a final concentration of 1U/ μl and incubating at 22°C for 15 min with mixing.

Lysate was bound to 100 μl Dynabeads Pan Mouse IgG (Thermo Fisher Scientific) for 30 min at room temperature. Beads were washed 3 times with 1 ml high salt wash buffer (30 mM HEPES pH7.5, 1M NaCl, 10% glycerol, 0.5% NP-40) then resuspended in 20 μl IP buffer + 50 U/μl RNase T1 and incubated at 22°C for 15 min before placing on ice. An additional three washes in high salt wash buffer were performed followed by a 1 ml wash in 1× Cutsmart buffer (NEB). Crosslinked RNA was dephosphorylated by resuspending the beads in 50 μl Cutsmart buffer with 0.5 U/μl CIP (NEB) and incubating at 37°C for 30 min with mixing. Beads were then washed twice with 500 μl 1× PNK buffer (NEB) then radiolabeled in 20 μl PNK buffer + 1 μl [γ-^32^P]ATP (0.5 μCi/μl final) (Perkin Elmer) + 1 U/μl T4 polynucleotide kinase (NEB) + 0.5 μl SUPERase.In (Thermo Fisher Scientific) at 37°C for 15 min before addition of a further 2 μl 10 mM ATP and further incubation for 10 min. Beads were then further washed 5 times with 1 ml PNK buffer without DTT (70 mM Tris–HCl pH 7.6, 10 mM MgCl_2_) then 25 μl (2 × NUPAGE + 1× NuPAGE sample reducing agent, Thermo Fisher Scientific) was added and samples were heated at 75°C for 10 min.

Crosslinked RBP complexes were separated on a 4–12% NU-PAGE bis-tris gel in 1× MOPS with 0.5 ml NuPAGE antioxidant in the upper chamber (0.5 ml in 200 ml, Thermo Fisher Scientific NP0005) at 4°C. Gels were transferred to Protran nitrocellulose in 1× NuPAGE transfer buffer with 10% methanol and 1 ml NuPAGE antioxidant. Radiolabelled RBP complexes were visualised by exposure to film overnight at –80°C. Correct sized bands were excised. RNA was isolated from nitrocellulose by incubation with 4 mg/ml proteinase K in 500 μl PK buffer (100 mM Tris pH 7.5, 50 mM NaCl, 10 mM EDTA) for 20 min at 37°C with mixing. A further 500 μl 7 M urea in PK buffer was added and samples were further incubated for 20 min at 37°C. RNA was then extracted with 1 ml phenol:chloroform:isoamyl alcohol (25:24:1)—pH 6.6 followed by ethanol precipitation. To prepare libraries, the TruSeq Small RNA Library Prep Kit (set A, Illumina) was used. Sample integrity was confirmed with a bioanalyzer (DNA 1000 kit – Agilent) before sequencing (Illumina HiSeq 4000 for Sro9/Slf1 samples, Illumina MiniSeq for Puf3).

### PAR-CLIP read processing

Details of the software versions used and their sources are given in Supplementary Table S10. Reads were trimmed using Trim Galore for the adaptor sequence (TGGAATTCTCGG) and TruSeq STP solution (CCACGTTCCCGTGG) using Trim Galore! Reads longer than 10 nt were mapped to *Saccharomyces cerevisiae* S288C reference genome R64-1-1 (sacCer3) using Bowtie with for settings -n 2 -v 2 -m 10 – best – strata. Sequencing read processing details for Slf1 and Sro9 are summarised in Supplementary Tables S1 and S2, Puf3 is in Supplementary Figure S2A.

PAR-CLIP binding sites were identified using PARalyzer (28) (v 1.5) with default settings except for the following: minimum read count per group = 5, minimum read count per cluster = 2, minimum read depth at a location to make a kernel density estimate = 5, minimum cluster size = 11 nt, minimum T-C conversion count for clusters = 2, minimum read length = 13 nt. Large clusters (>100bp) were filtered out to remove large spans of overlapping binding sites or rRNA contamination. Clusters were then matched to a BED file of features from Saccharomyces Genome Database (yeastgenome.org). Individual binding sites were defined as clusters of ≥5 overlapping sequencing reads of ≥13 nt with ≥2 T–C conversions per cluster and each binding site is ≥11 and ≤100 nt long. RNAs with at least one binding site were defined as targets.

### PAR-CLIP analysis

UTR lengths were compiled from several prior transcriptomics studies (29–33). The longest common variant was selected. tRNA-adaptation index (tAI) was from (34). Total RNA-Seq generated here (Supplementary Table S4) was used for mRNA abundance calculations. Ribosome profiling data were taken from (4). Translation initiation efficiency from (35). PARS data are from (36).

Motif discovery was done using DREME or MEME (37) using either a shuffled control set, conserving dinucleotide frequencies (DREME) or using a discriminative mode against non-targets (MEME). In each case default settings were used. The E-value quoted in the figure legend is the enrichment *P*-value times the number of candidate motifs tested.

For meta-analysis and comparison to ribosome profiling, single nucleotide mode binding positions from PARalyzer used the ModeLocation tool (ModeLocation = coordinate of the location with the highest signal/(signal + background) value). PAR-CLIP enrichments were calculated similarly to TE calculations for ribosome footprinting experiments: firstly, the total number of reads for a transcript from PARalyzer cluster analysis was divided by the sum-total PARalyser PAR-CLIP reads for that sample (to give reads per million). This was then divided by the coding sequence length in kb (to give reads per million per kb, rpkm). Enrichment is then calculated by dividing this rpkm for PAR-CLIP by the similar calculated rpkm from total RNA.

### Functional analysis

Functional analysis was performed using PANTHER (38) against a background of transcripts seen across all total samples using a statistical overrepresentation test for the Panther GO-Slim Molecular Function annotation data set. Multiple-testing correction was performed using a false discovery rate calculated using the Benjamini–Hochberg procedure (39).

### Total mRNA extraction

100 ml untagged or TAP-tagged cultures were grown to exponential phase (OD = 0.6) then split and half were treated for 15 min with 0.4 mM H_2_O_2_. This was performed in duplicate. RNA was extracted using 1 ml TRIzol (standard protocol - Thermo Fisher Scientific) and depleted for rRNA using Ribozero (Illumina). rRNA depletion and sample quality was assessed using a TapeStation (Agilent). Libraries were prepared using TruSeq stranded mRNA kit (Illumina) before sequencing on a Illumina HiSeq 4000. Reads were trimmed for the adaptors using TrimGalore and mapped to the R64-1-1 S288C sacCer3 Genome Assembly using Bowtie with standard settings. Mapped reads were counted using featureCounts. Differential expression was performed using DESeq2.

### mRNA polysome to monosome ratio analysis

Triplicate cell samples were grown and polysome fractionated as per (12) pooling fractions 4–8 (monosome) and 10–15 (polysome). Polysome vs monosomal enrichment of reads was performed using DESeq2 (supplementary Tables S5 and S6).

### Ribosome profiling analysis

Monosomal reads were taken from Wu *et al.* (4), (GSM3168396 and GSM3168403). Reads were trimmed with Cutadapt as per original study (NNNNNNCACTCGGGCACCAAGGA removed from 3′ and NNNN from 5′). Disome reads (40) (GSM4127880, GSM4127882, GSM4127886) were trimmed with Cutadapt using automatic settings. Reads were mapped to a non-coding and rRNA transcriptome then unmapped reads longer than 15 were mapped to the *Saccharomyces cerevisiae* S288C reference genome R64-1-1 (SacCer3). Monosome mapped reads were split into 20–22 nt reads (short footprints) and 28–30 nt (standard footprints). Disome reads were filtered for 57–63 nt reads.

Mapped reads were visualised using Integrative Genomics Viewer (IGV) (41). ORF mapped reads were then analyzed using geneBody_coverage.py using custom BED files comprising either all yeast ORF sequences (from www.yeastgenome.org), BED files of Slf1 or Sro9 target ORFs, or single nt position defined as the Slf1/Sro9 binding site mode (where mode = coordinate of the location with the highest signal/(signal + background) value ± upstream and downstream 100–500 bp. For analyzing ribosome density (TE), ribosome protected fragment read counts were normalised to rpkm values generated in this study (either stressed or unstressed as applicable).

### LacZ assay

YRE-LacZ assay was performed using strains GP7429, GP7431 and GP7433 as per (42). A minimum of three biological repeats were carried out for each strain.

### Serial dilution growth assays

Strains were grown from a starter culture until exponential growth phase (*A*_600_ = 0.6–0.7) then diluted to an OD of 0.1 and serially diluted in liquid medium. 2 μl each culture dilution was spotted onto solid medium ± hydrogen peroxide or copper sulphate. A minimum of three repeats were carried out for each strain.

### Protein expression analysis

Yeast (50 ml) were grown to OD = 0.4 before cultures were split and half were treated with 0.4 mM H_2_O_2_, cells were grown for 1–2 h. 10 OD units of culture were harvested by centrifugation, washed with 1 ml 10% TCA then resuspended in 200 μl TCA. 200 μl glass beads were added and cells were vortexed for 45 s at 4°C. A further 200 μl TCA was added and cells were vortexed again. Proteins were pelleted by centrifugation (10 min 16 000 × g 4°C), washed twice with 400 μl acetone, resuspended in 140 μl 100 mM Tris + 50 μl LDS sample buffer (Thermo Fisher Scientific) + 10 μl 2-mercaptoethanol and boiled at 95°C for 5 min. Proteins were then analyzed by SDS-PAGE and western blot. Quantification was done relative to WT untreated and normalised to a loading control for three replicates. For eIF2α-Phosphorylation determination, Pierce Protease and Phosphatase Inhibitor Mini Tab (ThermoFisher scientific) was added during the lysis of log phase yeast cells (at time = 0 and 15 min after addition of 0.8 mM H_2_O_2_).

### SDS-PAGE and western blot

Protein separation was performed using either 4–12% Bis–Tris or 10–20% Tris-glycine gels. Gels were transferred to Protran nitrocellulose (Sigma) and probed with either custom antibodies or commercial primary antibodies followed by Licor secondary antibodies. Westerns were developed and quantified using a LI-COR Odyssey® CLx Imaging System. Where indicated, Revert™ 700 Total Protein Stain Kit for Western Blot Normalization (Li-Cor Biosciences) was used.

### eIF4E-TAP immunoprecipitation

Two liters GP6001, GP5997 and GP8120 were grown in triplicate to an OD of 0.6 in SCD media then split into 2 × 1 l flasks. One of these was treated with 0.4 mM hydrogen peroxide. After 15 min further growth, cells were harvested by centrifugations, frozen and ground under liquid Nitrogen in a cryogenic mill. Ground cells were resuspended in 1 ml IP buffer (30 mM HEPES pH7.5, 100 mM KCl, 10% glycerol + protease inhibitors) and clarified at 20 000 × g. 100 μl lysate was for sample inputs, the remainder was was bound to 300 μl Dynabeads Pan Mouse IgG (Thermo Fisher Scientific) for 30 min at room temperature. Beads were washed 5 times quickly with 1 ml IP buffer followed by two 1 ml washes (30 min at room temperature) with the addition of 0.5% NP-40. Beads were then resuspended in 275 μl IP buffer. 25 μl was boiled with sample loading buffer (2 min) for western-page analysis, the remainder was RNA extracted using 0.75 ml TRIzol™ LS Reagent (standard protocol) and depleted for rRNA using Ribozero (Illumina).

### Polysome fractionation

Cells grown in triplicate to an OD600 of 0.7 were crosslinked with 1 mg/ml cycloheximide (with and without oxidative stress (15 min incubation at 30°C with 0.4 mM H_2_O_2_). The pelleted cells were dissolved in lysis buffer (10 mM Tris pH 7.4; 10 mM MgCl_2_; 250 mM KCl; 25 mM EGTA; 1 mM DTT) and were vortexed with 700 μl chilled glass beads in a 15 ml culture tube circular base for 6–7 times in a slanting position for 40 seconds in the cold room, with 60 s intervals. After a rapid centrifugation (5000 × g), the cellular components, i.e. supernatant, were transferred to a microcentrifuge tube (Eppendorf), and was centrifuged briefly at 0°C, 13000 x g to remove any residual beads or cell debris.

*A*_260_ = 3 units of cell lysate was loaded onto a 12 ml 10–50% sucrose gradient in buffer (100 mM Tris Acetate pH 7.4, 700 mM Ammonium Acetate, 40 mM Magnesium Acetate, DEPC water) and in open-top polyallomer tubes (Seton Scientific) and ultracentrifuged at 4ºC for 2.5 h at 40 000 rpm in a Beckmann SW41 rotor. Following ultracentrifugation a total of five fractions were collected using a gradient fractionator (Isco Brandel). The *A*_254_ signals were collected with VI logger data logger software (National Instruments) and plotted as traces using MS excel. Fractions obtained from polysome profiling were subsequently processed for RNA analysis as described below.

### qPCR

RNA extraction using TRIzol. TRIzol reagent was added equal to half the volume of each fraction (polysome or IP or total soluble cell extract) along with chloroform to separate the aqueous phase containing RNA from rest cellular components in organic phase by centrifuging at 4°C for 15 min at 13 000 rpm. The RNA was precipitated with one volume of isopropanol, 4 μl glycoblue and spiked in Promega luciferase control RNA (2 ng Luc/1 ml fraction) at –80°C overnight and centrifuged next day for 15 min at 4°C at 13 200 rpm. The RNA pellet was washed with 100% ethanol and air dried at 37°C for 5 min. The RNA was resuspended in 20 μl Nuclease free water.

The extracted RNA was treated with RNase free DNase (Promega) to remove any DNA contamination, and the quality was assessed (Nanodrop ND8000 spectrophotometer). 0.5–1 μg of RNA was used to synthesis cDNA using ProtoScript® II First Strand cDNA Synthesis Kit (NEB) with the manufacturers protocol. The cDNA obtained was used in qPCR reactions with 300 mM gene-specific primers (Supplementary Table S9). CFX Connect Real-Time PCR Detection System was used to obtain Cq values of the samples and results were quantified using delta-delta Ct method with spiked in luciferase RNA as the control. The Pfaffl method was used to calculate primer-pair amplification efficiencies (43). Briefly, a cDNA 1:10 dilution series was used to construct a standard curve with at least four points, the first of which was an undiluted cDNA sample. The computed slope was then entered into the formula: efficiency% = (10^(–1/the slope value)^ – 1) × 100 to calculate the percentage of primer efficiency and the amplification factor used to adjust Ct values in qPCR analyses (see also: https://toptipbio.com/calculate-primer-efficiencies/). The mean% RNA per fraction ± sem (*n* = 3) was plotted to compare selected RNA migration following stress in WT and *slf1Δ* strains(44,45). A two-way ANOVA with a Tukey post hoc test was used to test whether differences in relative mRNA abundance in any fraction were due to (i) the strain: *slf1Δ* versus WT, (ii) the growth conditions: optimal versus stress or (iii) an interaction between strain and growth conditions. *P* < 0.05 was used as a cut-off for statistical significance.

### Disome profile analyses

Cell lysates were prepared using standard protocol (as above) in triplicate and RNase1 (2.5 U/*A*_260_) (Invitrogen) was added and lysates were incubated with agitation at 37°C for 30 min on a benchtop thermal incubator shaker (Thermofisher), before 1 μl RNase inhibitor (Invitrogen) was added. The treated lysate was layered on top of a 10–35% sucrose gradient and ultracentrifuged for 2.5 h at 40 000 rpm, 4°C in an SW41 rotor (Beckmann). *A*_260_ trace signals were captured with data logger software as described above. Fractions collected from disome profiling experiment with the *SLF1::TAP* strain were processed for protein analysis using western blotting. 750 μl Each fraction was treated with 40% TCA and precipitated overnight. Following centrifugation for 12 000 rpm for 15 min at 4°C. The supernatant was discarded, and the pellets were twice washed with cold acetone. After air drying pellets at room temperature for 20 min, they were resuspended in 2× loading dye (1 M Tris (not-pH adjusted)). After heating the samples at 95°C for 5 min, they were centrifuged briefly and the supernatant was collected, while any remaining contaminated pellets were discarded. The extracted proteins were run in Novex NuPAGE™ 4 to 12%, Bis–Tris Gels with 1× NuPAGE MOPS SDS Running Buffer using XCell SureLock System (Thermo Fischer) and transferred to nitrocellulose membrane. To visualise proteins in monosome and disome fractions, the membranes were incubated with primary antibodies (α-Prot-A for TAP tag, α-RPS3 followed by secondary rabbit antibodies (LI-COR). Signals were quantified using LI-COR software.

### Dual luciferase reporter (DLR) assays

Log phase yeast cells were harvested (unstressed and after 2 h of treatment with 0.8 mM H_2_O_2_), and total protein was extracted using the acid-washed glass bead method to obtain cell lysate in 1× ice-cold passive lysis buffer (PLB) (DLR Kit, Promega), supplemented with Pierce Protease Inhibitor Mini Tablets (Thermo Fisher Scientific). LAR II and Stop-and-Glo® Reagent were prepared using the DLR Kit and DLR assay was performed according to the manufacturer's instructions (Promega) using a GloMax 20/20 Luminometer (Promega). The measurements for each strain were taken in triplicate. Statistical analysis was done in GraphPad Prism (GraphPad Software). For each strain the ratio Fluc/Rluc was used to calculate the %readthrough for each stop codon reporter UAA, UAG and UGA normalised to the CAA codon reporter (46). Similar analyses were carried out to calculate % frameshift for all strains with the –1 and +1 reporter plasmids (47). DLR ratios were normalised to the ratio of the frame 0 reporter for the strain and condition.

### Reagents

All key reagents and product numbers are listed in Supplementary Table S10.

### Data availability/sequence data resources

Sequencing files are deposited with the GEO accession number GSE174707.

### Statistical analyses

The test type used, number of replicates and resulting significant *P* values are all given in the legend to each figure.

### Data availability/novel programs, software, algorithms

No novel software was generated as part of this study.

### Web sites/data base referencing

Websites and programs used in the manuscript are listed under specific methods above and in the key resources table (Supplementary Table S10).

## RESULTS

### Sro9 and slf1 bind within open reading frames of ribosomal protein and stress response genes

**Figure 1.**
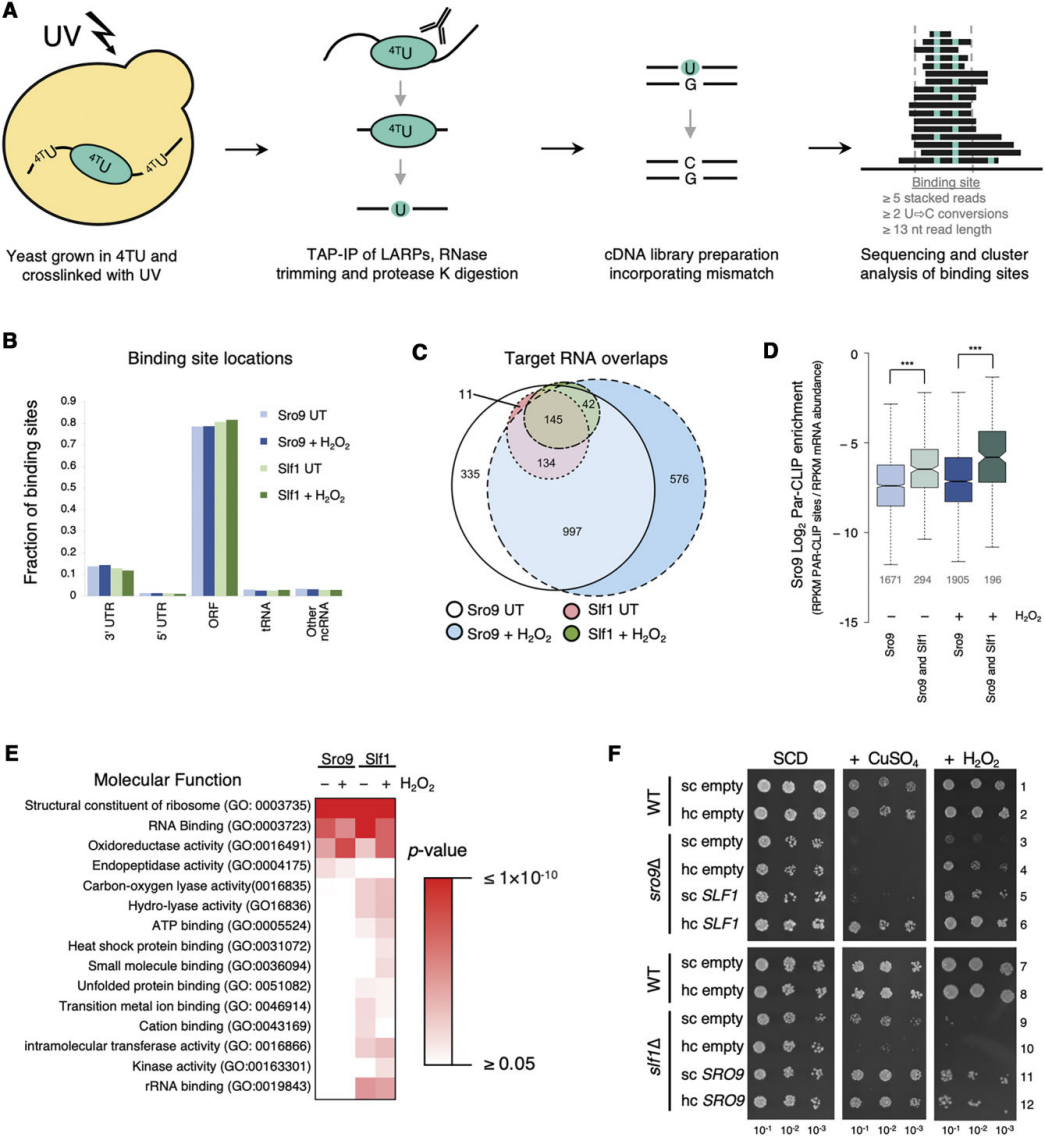
Sro9 and Slf1 bind in mRNA coding regions. (**A**) Overview of PAR-CLIP protocol. (**B**) Distribution of Sro9 and Slf1 PAR-CLIP binding sites from 0.4 mM H_2_O_2_ treated or untreated (UT) yeast. Other ncRNA includes CUT, SUT, ncRNA, snRNA, snoRNA and rRNA. Shades of blue and green are used for Sro9 and Slf1, respectively with darker shades for H_2_O_2_ treated. (**C**) Overlap between target mRNAs identified in PAR-CLIP studies, segment numbers >5 are indicated. (**D**) PAR-CLIP enrichment of Sro9 targets. Enrichment of PAR-CLIP rpkm/RNA-Seq rpkm for each mRNA in a group, comparing all Sro9 targets and the subset also bound by Slf1. Boxes extend from 25–75% of the data range with notches around median. The notches are ±1.58 × interquartile range(IQR)/sqrt(*n*) and represent the 95% confidence interval for each median. Whiskers extend to data points that are less than 1.5 x IQR away from 1st/3rd quartile. The number of mRNAs in each group is given below each plot (grey). *P*-values are Mann–Whitney test (*** left to right = 4.273e–15, < 2.2e–16). All Sro9 colouring as in panel B, Slf1 shared genes have lighter shaded boxes. (**E**) Functional categorization. Sro9 and Slf1 bound transcript Go-slim ‘Molecular Function’ over-enriched terms either in the absence (−) or presence (+) of H_2_O_2_. *P-*values are Fisher's Exact test corrected for false discovery rate. (**F**) Phenotypic serial dilution growth assay complementation of deletion strain phenotypes with single copy (sc) or high copy (hc) plasmids. ‘empty’ denotes controls plasmids without *SLF1* or *SRO9*. Growth medium is synthetic complete (SCD) with or without 2 mM CuSO_4_ or 1.8 mM H_2_O_2_. Top: wild-type (WT), *sro9Δ* and its complementation with *SLF1*. Bottom: WT, *slf1Δ* and its complementation with *SRO9*.

To investigate how Slf1 and Sro9 affect translation we performed Photoactivatable Ribonucleoside-enhanced Crosslinking and Immunoprecipitation (PAR-CLIP) experiments in both actively growing cells and following a short (15 min) oxidative stress (48). In our approach (Figure 1A), unstressed or peroxide stressed strains bearing genome integrated Slf1-TAP or Sro9-TAP C-terminal tags were used. These cells behave like WT cells, preserving the major translational control response to oxidative stress via increased phosphorylation of eIF2α mediated by Gcn2 (Supplementary Figure S1B) (1). Duplicate cell cultures were grown for each condition in the presence of 4-thio uracil (4TU) and UV crosslinked prior to isolating the ribonucleoprotein complexes via the common TAP tag (Figure 1A). RNA trimming and cDNA conversion causes 4TU-G mismatch base pairing at the cross-linking site, that is observed following genome mapping of reads as a T-to-C conversion (Figure 1A). Binding sites for each protein were identified through clustering analysis (28). Non-cross-linked IP controls confirmed specificity of cross-linking for the tagged proteins (Supplementary Figure S1C). Sequencing was performed on total and PAR-CLIP RNA (sequencing read and processing information is summarised in Supplementary Tables S1 and S2). As an independent control, we performed parallel studies of the RNA binding protein Puf3 that has a well-defined set of target mRNAs (12,49) and consensus binding motif typically found within the 3′UTR (50). Our Puf3 PAR-CLIP analyses faithfully recapitulated previous findings showing that Puf3 PAR-CLIP sites (i) preferentially enrich 3′UTRs and overlap 3′ end of ORFs (Supplementary Figure S2A–D), and (ii) feature the expected Puf3 binding consensus sequence (Supplementary Figure S2E). These controls suggested our methods were suitable and appropriate.

Most LARP binding sites (defined as clusters of overlapping PAR-CLIP reads in Figure 1A) were found within open reading frames (ORF) of mRNAs (Figure 1B, Supplementary Table S3), independent of whether cells were stressed. Around 12% of sites were mapped within 3′UTRs and fewer within 5′UTRs. RNAs with at least 1 binding site were defined as a target of that LARP, although typically 3–5 separate binding sites were identified per bound mRNA (Supplementary Figure S3A). Thus, a standard bound mRNA has several binding sites within the ORF with some possessing additional binding sites in their 3′UTR (Supplementary Figure S3B). Sro9 bound a much wider set of mRNAs than Slf1, likely reflecting the proteins relative abundance. From western blotting of TAP-tagged proteins we estimate Sro9 is approximately 13.5 times as abundant as Slf1 (Supplementary Figure S3C). Sro9 target mRNAs included all Slf1 targets in unstressed cells and almost all following the 15-min peroxide stress (Figure 1C). By calculating relative enrichment of Sro9 PAR-CLIP reads to our total RNA-Seq reads we determined that Sro9 was more highly enriched on the subset of mRNAs that also bound Slf1 compared with those binding Sro9 only (Figure 1D), suggesting the shared mRNAs are the mRNAs favored by both proteins and that the more abundant Sro9 can additionally bind other mRNAs. Targets are enriched with mRNAs encoding ribosomal proteins and oxidoreductases independent of peroxide treatment (Figure 1E). This was in good agreement with mRNAs enriched in prior RIP-seq or RIP microarray studies (Figure 1E, Supplementary Figure S3D and E) (24,25). We conclude that our PAR-CLIP approach has identified the main mRNA targets of both LARPs, that Slf1 binds a sub-set of Sro9 targets, and that the mRNA target identities are not dramatically altered by stress.

The large target-overlap between LARPs suggests that they have a common role. Deletion of either gene (*sro9*Δ or *slf1*Δ) gives rise to similar oxidative stress-related phenotypes (Figure 1F, rows 3 and 9), suggesting that gene dosage may be critical for normal stress responses (24,25). We performed cross-complementation studies by transforming the single deletion strains with the opposite LARP gene on either single copy (sc) or high-copy (hc) plasmids. In addition to peroxide, we also tested copper sensitivity as both proteins were initially characterised with roles in copper homeostasis (51). The *sro9*Δ phenotypes were complemented by *SLF1* on a hc plasmid (Figure 1F, compare rows 3–6). *slf1*Δ phenotypes were complemented by sc or hc *SRO9* (Figure 1F, rows 9–12). As Sro9 levels are significantly higher than Slf1, these data fit well with the idea that both proteins are largely targeting and can function similarly on the same mRNAs and that cells are tuned to the total expression levels of both the LARPs.

### LARP binding sites are enriched towards ORF 3′ ends of abundant, efficiently translated mRNAs

The LARP proteins interact with transcripts having significantly short 5′UTRs. They have distinct ORF lengths, as Slf1 targets are short while the larger set of Sro9-bound mRNAs are longer on average than non-targets (Supplementary Figure S4A-C). Targeted mRNAs are among the most abundant (Supplementary Figure S4D) and are enriched for optimal tRNA use, as measured using the tRNA-adaptation index (tAI) (Supplementary Figure S4E) (34). In line with these observations, using condition-appropriate ribosome profiling datasets (4) and our own matched RNA-seq experiments (Supplementary Table S4) to determine the density of ribosomes (also known as translation efficiency or TE) under both growth conditions showed that significantly more ribosomes were associated with mRNA targets of either LARP than non-targets (Supplementary Figure S4F). The calculated efficiency of translation initiation was also significantly enhanced for these mRNAs (Supplementary Figure S4G). As the target mRNAs are heavily enriched for ribosomal protein transcripts which exemplify all these characteristics, we repeated the above computational analyses after removing this group of mRNAs. The only impacts were to eliminate the significance of 5′UTR lengths and the preference of Slf1 for shorter ORFs (data not shown). In summary, the mRNAs bound by the LARP proteins encode ribosomal proteins and a subset of other abundant transcripts that have optimal codons and are highly translated and ribosome-dense.

**Figure 2.**
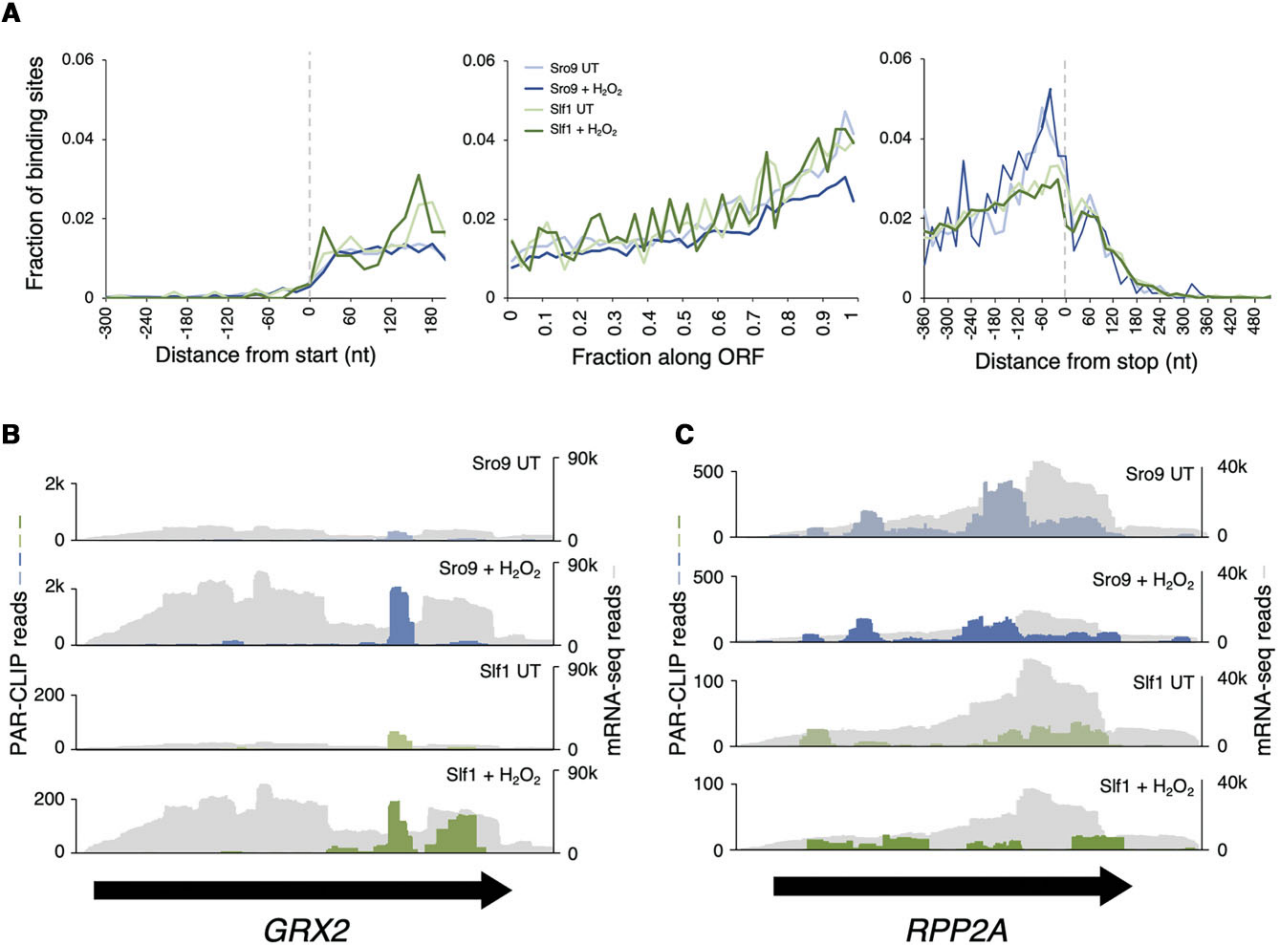
Sro9 and Slf1 binding increases along ORFs. (**A**) Aggregate ‘metaplot’ of mRNA binding site distribution from PAR-CLIP relative to start codons (left), stop codon (right) measured in nucleotides (nt) and across the ORF (middle) where each ORF is compressed into 100 centiles for Sro9 and Slf1 from H_2_O_2_ treated or untreated (UT) yeast coloured as in Figure 1B. (**B** and **C**) PAR-CLIP coverage on example mRNAs: (B) *GRX2* and (C) *RPP2A*. RNA-seq coverage in grey, PAR-CLIP coloured as in Figure 1B. Black arrows indicate the extent of each coding region.

In contrast to our total RNA controls, meta-analysis of LARP binding site positions using PARalyzer where each binding site is equally weighted (28) (Figure 2A), or by mapped read density level (Supplementary Figure S5A) demonstrates a clear bias towards ORF 3′ end binding in all four datasets. In contrast, meta-plots of condition-appropriate ribosome profiling data (4) confirmed 80S ribosomes distribute evenly along LARP-target ORFs (Supplementary Figure S5A). For individual mRNAs, there are CLIP sites shared by both factors e.g. in antioxidant enzyme mRNAs *GRX2* (Figure 2B), *TSA2* and *TRX2 (*Supplementary Figure S5B). While at other mRNAs the CLIP site patterns differ between LARPs (e.g. *RPP2A*, Figure 2C). Taken together these PAR-CLIP patterns, combined with global ribosome footprinting data and proteomics measurements indicating LARPs are significantly less abundant than ribosomes (52), are consistent with the idea that LARPs bind ORFs in the vicinity of a subset of translating ribosomes.

### Slf1 impacts ribosome loading onto its targets without moderating eIF4F recruitment

*slf1Δ* cells are more sensitive to peroxide than *sro9*Δ or WT cells. By several measures we found that the Yap1-mediated oxidative stress induction of antioxidant enzyme mRNAs is maintained in the *slf1*Δ strain. This included activation of a Yap1-dependent *lacZ* reporter, RNA-seq analyses of transcriptomes and qRT-PCR experiments of specific mRNAs (Supplementary Figure S6A–C, Supplementary Table S5). We also confirmed by western blotting that three antioxidant enzymes for which antibodies were available (Zwf1, Tsa1 and Trx2) are induced by stress in WT and *sro9*Δ cells. Interestingly, this was not the case in *slf1Δ* cells (Supplementary Figure S6D). These analyses demonstrate that Slf1 loss has greater impact on the stress response than Sro9 and that it is acting post-transcriptionally. Therefore, we focused most of the following described analyses on Slf1.

As the mapped CLIP sites appear accumulated upstream of stop codons, we used a well-established dual luciferase bicistronic reporter (DLR) system to evaluate whether stop-codon recognition was altered by loss of Slf1 (46). In this assay the firefly luciferase ORF is placed after the stop codon of the *Renilla* ORF so that it is only expressed by mis-recognition of the stop codon as a sense codon, thereby creating a fusion protein with both luciferase activities. We found that stop codon readthrough rates with these reporters were not impacted either by oxidative stress or by loss of Slf1 (Supplementary Figure S7A). We concluded that neither oxidative stress nor Slf1 was having a general impact on stop codon recognition.

**Figure 3.**
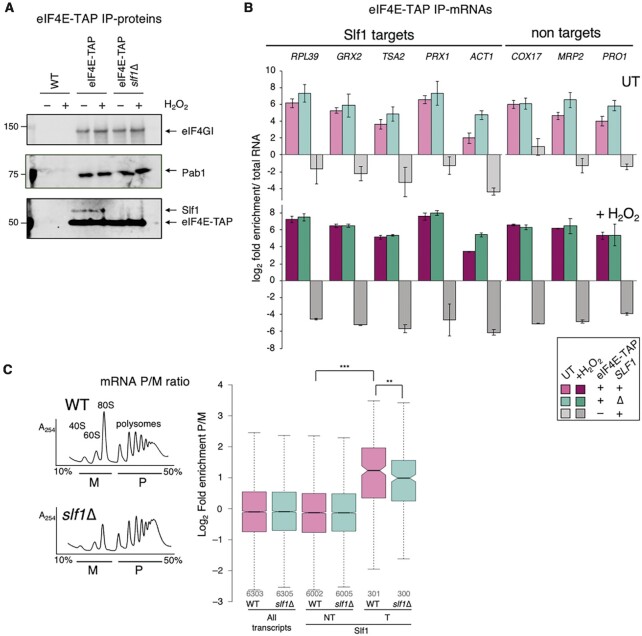
No reduction in eIF4E-mRNA interactions in *slf1Δ*. (**A**) Western blots showing eIF4F factor association with eIF4E-TAP in the presence or absence of Slf1. (**B**) qRT-PCR of mRNAs associated with eIF4E-TAP ± Slf1 ± H_2_O_2_. *ACT1* is in ‘Costello group I’, all other targets are in IIIA. Non-targets: group IVA (*COX17*, *MRP2*) or IVC (*PRO1*) (*n* = 3). Samples coloured as indicated in boxed key (magenta for WT and cyan for *slf1*Δ cells, with darker shades for ±H_2_O_2_). (**C**) Polysome profiles (*A*_254_ traces from 15–50% sucrose gradients) of extracts from wild-type (WT) and *slf1*Δ cells. Underlined areas marked monosome (M) and polysome (P) were pooled for mRNA-seq (*n* = 3). Right, Ratio of polysome:monosome association for indicated grouped mRNAs in wild-type (WT) and *slf1*Δ strains. Box plot parameters explained in legend to Figure 1, colours as panel B. *P*-values shown are *** < 2.2 × 10^−16^ and ** = 0.00350 (Mann–Whitney test).

Efficient recycling of ribosomes released at stop codons could help ribosomes initiate again rapidly on the same mRNA, and such a role was proposed for LARP1 (53). Ribosome recruitment to mRNAs relies on the eIF4F complex binding to the 43S pre-initiation complex. Differences in mRNA association with translation factors that interact with the 5′ cap and/or polyA tail were observed previously in RIP-seq experiments (54). mRNAs were clustered into seven groups based on eIF4F and eIF4E binding protein (4E-BP) enrichment profiles (Supplementary Figure S7B). We find LARP target mRNAs are predominantly members of groups I and IIIA. Group I (including oxidoreductase mRNAs) are relatively depleted for eIF4F, while Group IIIA (encoding ribosomal structural and biosynthetic proteins) were termed the ‘strong closed-loop’ group (55) and are enriched with both eIF4E and eIF4G, but exclude the 4E-BPs (54). To test if Slf1 does impact eIF4F recruitment at the 5′ end we performed RIP with eIF4E-TAP in *slf1Δ* cells or otherwise WT cells in the absence or presence of peroxide. We analyzed protein associations via western blotting (Figure 3A) and selected target and non-target mRNAs by qRT-PCR (Figure 3B). Although we confirmed that we captured ∼95% of total eIF4E in a tag-specific manner (Supplementary Figure S7C), these experiments revealed no clear change in eIF4E-protein interactions or eIF4E-mRNA associations following loss of Slf1 (Figure 3A and B). Hence, we conclude that the translation defect must occur at a step following mRNA recruitment and before translation termination.

We performed polysome profiling (Figure 3C) and collected mRNA from fractions of both WT and *slf1Δ* cells and processed them for differential RNA-seq analyses of polysome (P) or monosome (M) fractions (Supplementary Table S6). Here, we wanted to evaluate relative ribosome engagement of target and non-target mRNAs. Slf1 CLIP target mRNAs were significantly enriched in the P fractions compared to the M fraction when compared to non-targets in WT cells, consistent with their efficient translation (Supplementary Figure S4F). Deleting *SLF1* partially diminished the polysome enrichment of these targets, but ribosomes remained well-engaged. Together these experiments suggest that ribosomes can be recruited efficiently to Slf1 target mRNAs in *slf1*Δ cells.

**Figure 4.**
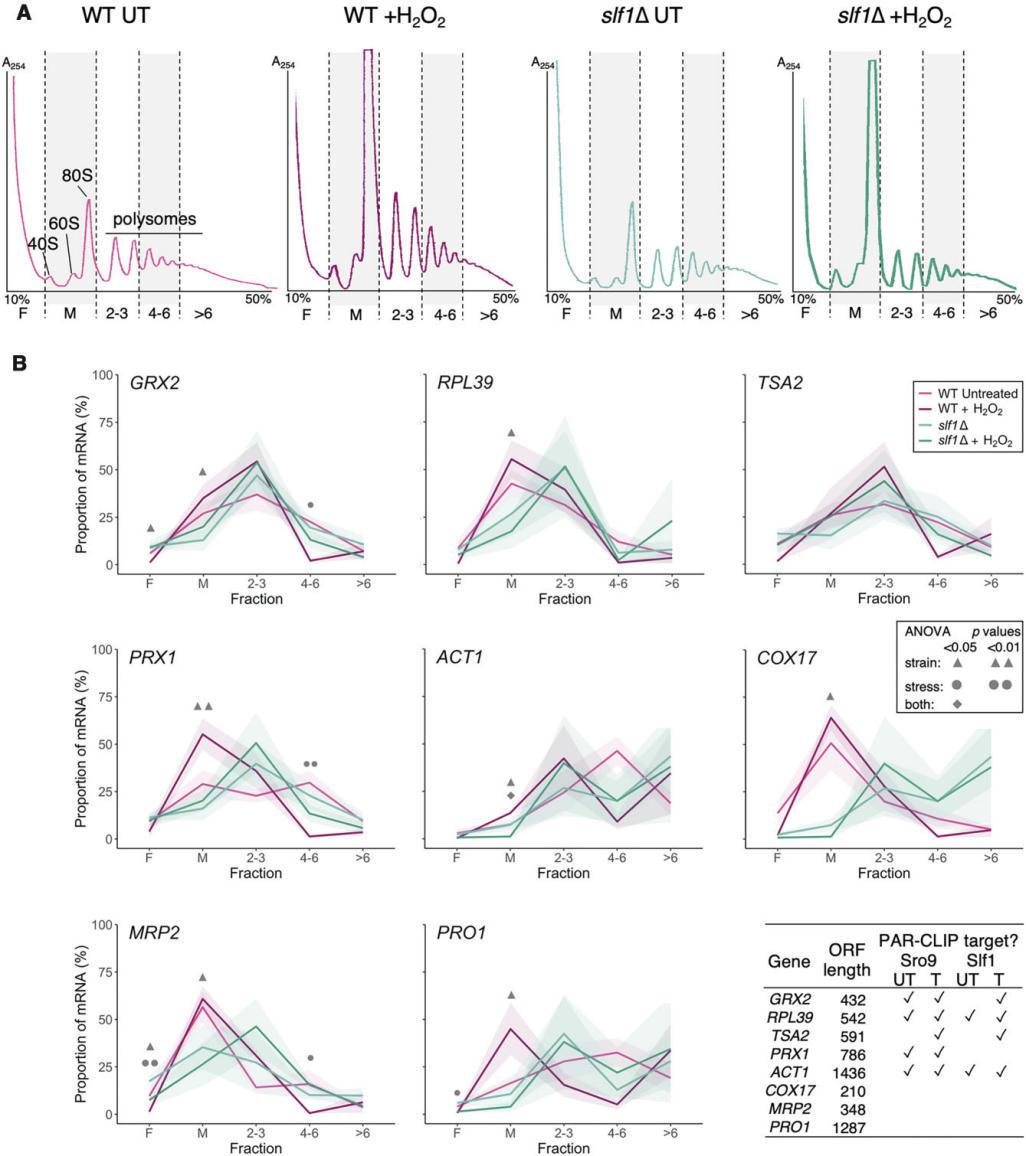
mRNAs remain ribosome associated during stress in *slf1Δ* cells. (**A**) Polysome profiles from 15–50% sucrose gradient fractionated cell extracts with stripes indicating five pooled fractions collected for qRT-PCR: F = ribosome free, M = monosome 2–3/4–6/>6 = increasing polysome association. Traces coloured as per key in panel B. (**B**) Proportion (%) of individual mRNAs found in each polysome fraction by qRT-PCR in WT (magenta) or *slf1Δ* (cyan) in optimal growth conditions or following 15 min H_2_O_2_ treatment (darker shades). The shaded uncertainty envelope around each line represents the s.e.m. (*n* = 3). A two-way ANOVA with a Tukey post hoc test was used to assess the effect of the strain and the stress and to test the interaction of these two factors for each gradient fraction. Symbols (defined in the inset key) indicate significant results. Table shows which RNAs are PAR-CLIP targets in each dataset (✓).

To extend these analyses and include stress, we repeated polysome gradient fractionation and collected gradient fractions from which we analysed the migration of specific Slf1 target and non-target mRNAs by quantitative reverse transcription PCR (qRT-PCR). As expected from prior experiments, oxidative stress caused ribosome-run off revealed by reduced polysomes and increased 80S peak height which was partially exacerbated by *slf1Δ* (Figure 4A). We partitioned gradient eluates into five fractions and performed qRT-PCR for five Slf1 target mRNAs and three non-target mRNAs with various ORF lengths (Figure 4B). In unstressed cells the fraction with the greatest proportion of mRNA (peak fraction) typically correlated with ORF length. In WT cells, stress shifts the mRNA patterns to lighter sucrose fractions so that the mRNAs peak in the M or the 2–3mer fractions (Figure 4B, light and dark pink) in line with the global shift in the rRNA A_254_ trace caused by eIF2α phosphorylation (Figure 4A).

In *slf1Δ* cells the patterns differ. There is a modest increase in the amount of free mRNA (fraction F), for some mRNAs (e.g. *TSA2*, *PRX1*, light green) in line with the previously observed shift of some heavier polysomal mRNA to lighter fractions in our lower-resolution polysome-seq data (Figure 3C). Slf1-target mRNA peaks center in the 2–3mer fraction, especially following stress (Figure 4B, dark green). In summary, individual mRNAs are maintained on polysomes in the *slf1Δ* strain including during stress. These data in Figures 3C and 4B indicate that the failure to induce antioxidant proteins in stressed *slf1*Δ cells is not likely caused by an inability to recruit ribosomes to the mRNA and initiate translation. This implies that Slf1 does not play a critical role in recruiting ribosomes to mRNAs, instead the data as a whole, suggest Slf1 is acting after ribosome recruitment and prior to translation termination, for example during elongation.

### Sro9 and slf1 binding sites are enriched for ribosomes

**Figure 5.**
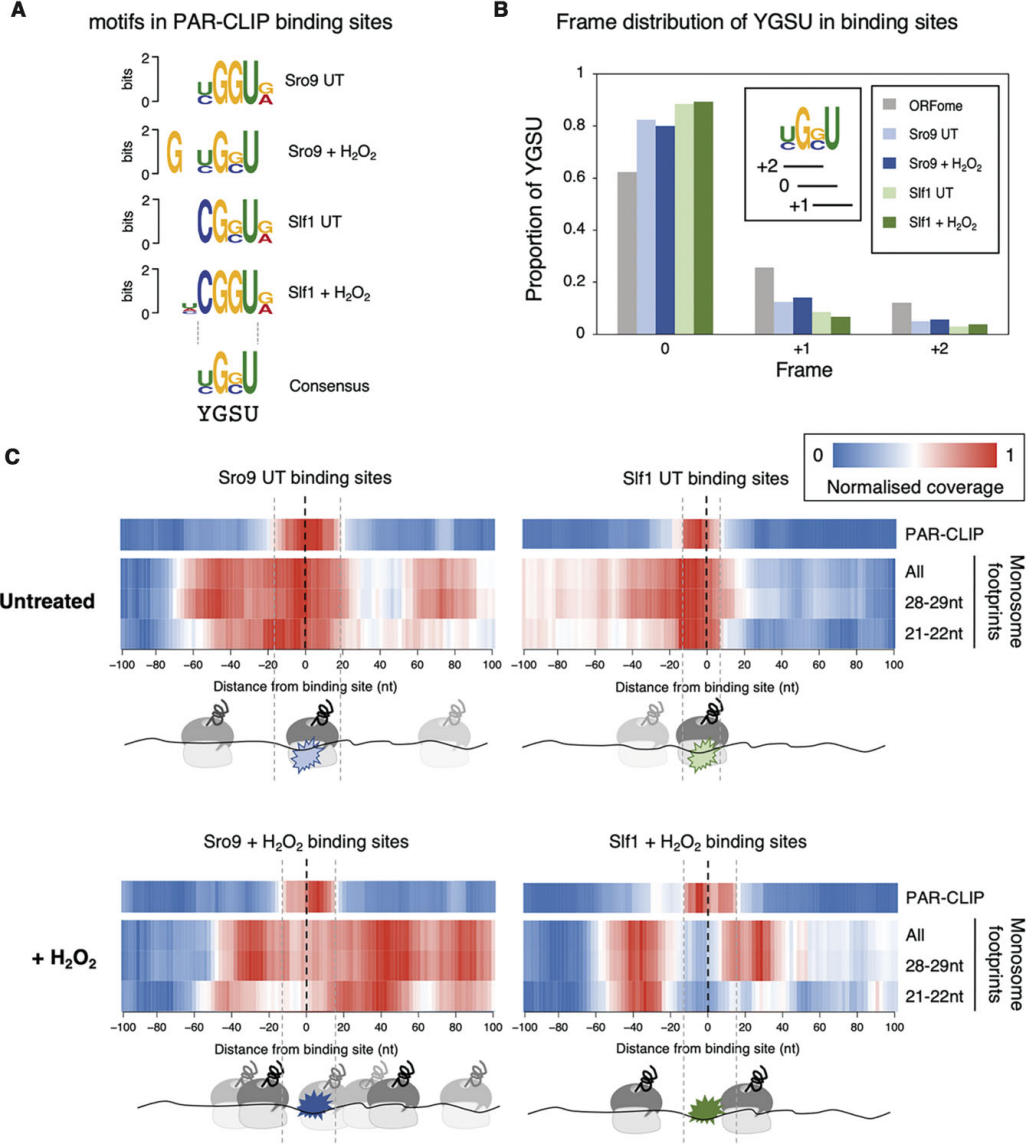
LARP binding sites align with ribosome footprints. (**A**) The most enriched motif identified within PAR-CLIP mRNA binding sites in each condition, using DREME, aligned around the common GSU triplet. (Motif *E*-values (top to bottom): 1.1 × 10^−98^, 6.0 × 10^−153,^1.2 × 10^−30^, 4.5 × 10^−16^). (**B**) Reading frame distribution of GSU framing within PAR-CLIP binding sites and within ORFome, colouring as figure 1B. Inset shows the 0, + 1 and + 2 framing with respect to YGSU. (**C**) Ribosome read density around PAR-CLIP binding site mode locations (Supplementary Table S3) (-100 upstream to + 100 nt downstream). Plots show normalised sequence read coverage (enriched red) from either UT (top) or stressed (+ H_2_O_2,_ bottom) conditions. Aligned PAR-CLIP reads for Sro9 (left) and Slf1 (right) cluster around 0. 80S monosome ribosome reads from a recent published study (4) mapping within each 200 nt binding site window are shown below each plot for standard ribosome footprints (28–29 nts) or small rotated ribosome footprints (21–22 nts) representing rotated ribosomes with free A-sites and both of these sets combined (All). Cartoons below each plot indicate relative positions of each LARP and the apparent preferred ribosome positions.

We examined our PAR-CLIP data for motifs and found short sequences in common (Figure 5A). An enrichment of G and U and a YGSU consensus present in ∼50% of ORF binding sites, some of which contained multiple motif copies (Supplementary Figure S8A). In > 80% of binding sites containing the motif, the consensus G was in the same frame. Hence motif GGU or GCU triplets almost always code for glycine or alanine, respectively (Figure 5B). There is reading frame bias throughout the yeast ORFome favoring this skewed distribution due to codon usage and di-codon bias, but it is more extremely biased at these PAR-CLIP sites. GGU and GCU codons are not predicted to be decoded slowly (56); their decoding tRNAs are both abundant (Supplementary Figure S9A). We obtained tRNA cross-links in our PAR-CLIP data, including to tG(GCC) that recognises GGU, but not to tA(AGC) that binds GCU (Figure 1B, Supplementary Figure S9B). The YGSU and tRNA enrichments were stress-independent and remain unexplained.

The reading frames of the leading and trailing edges of PAR-CLIP sites revealed a frame bias (Supplementary Figure S8B) that is maintained over different footprint sizes (Supplementary Figure S8C) suggesting that the proximity of ribosomes was influencing RNase T1 mRNA trimming. The presence of tRNA reads among CLIP data also suggests close proximity of the LARPs to ribosomes (57). We used mapped positions of 80S ribosomes identified through an independent recent ribosome profiling study performed both in optimal growth conditions as well as following oxidative stress with peroxide to assess relative ribosome and LARP locations (4). Wu and colleagues quantified two ribosome footprint sizes; small (20–22 nt) footprints corresponding to rotated ribosomes with open A-sites and standard (28–29 nt) A site occupied footprints. We separately compared the positions of small and standard footprints with our PAR-CLIP datasets. No clear difference was observed between the metaplots of total ribosome distribution across our targets ORFs compared to all transcripts (Supplementary Figure S10A). The 3′ ORF bias we observe in PAR-CLIP reads was not mirrored by the total ribosome distribution (compare Supplementary Figures S5A and S10A).

We compared the positions of ribosomes to our PAR-CLIP sites by centering the PAR-CLIP positions (mode locations) at 0 and assessing where ribosomes accumulate within 100 bases upstream (towards the start) or downstream (towards the stop) (Figure 5C). In all cases we observed a clear enrichment of ribosomes at or close to LARP binding sites. In untreated conditions, Slf1 sites in particular are enriched for a single ribosome co-incident with the mapped Slf1 binding sites (Figure 5C, top panels), Sro9 sites show a similar pattern, but additionally enrich ribosomes spaced downstream (nucleotides (nt) +60 to +90) and upstream (nt –40 to –70) of the CLIP sites. The same trends are observed when increasing nt length windows around each CLIP site (up to ±500 nt; Supplementary Figure S10B). The relative depletion of ribosomes ahead of Slf1 footprints may be in part related to CLIP site enrichment before stop codons (Figure 2A). Under peroxide treated conditions, the ribosome patterns shift relative to the CLIP sites. For Slf1 the pattern shows two peaks of ribosome enrichment, one in downstream and one upstream of the CLIP site, suggesting Slf1 here sits between ribosomes. The stressed ribosome pattern at Sro9 CLIP sites is less clear but retains pronounced stripes of ribosome enrichment (coloured red) either side of the Sro9-bound sequences. The shorter 21–22 nt footprints, indicative of empty A-site rotated ribosomes (4), only accumulate behind the Slf1 CLIP positions and mainly in front of Sro9 CLIP sites revealing a potential distinction between the factor binding preferences. In conclusion these analyses suggest that the LARPs may interact with targeted mRNAs as ribosome–LARP complexes in unstressed cells, while the factors appear to shift to mRNA between spaced ribosomes during stress. In effect, many of the mapped CLIP sites may represent ‘selective’ ribosome footprints.

### Slf1 binds to disomes and prevents programmed ribosome frameshifting

**Figure 6.**
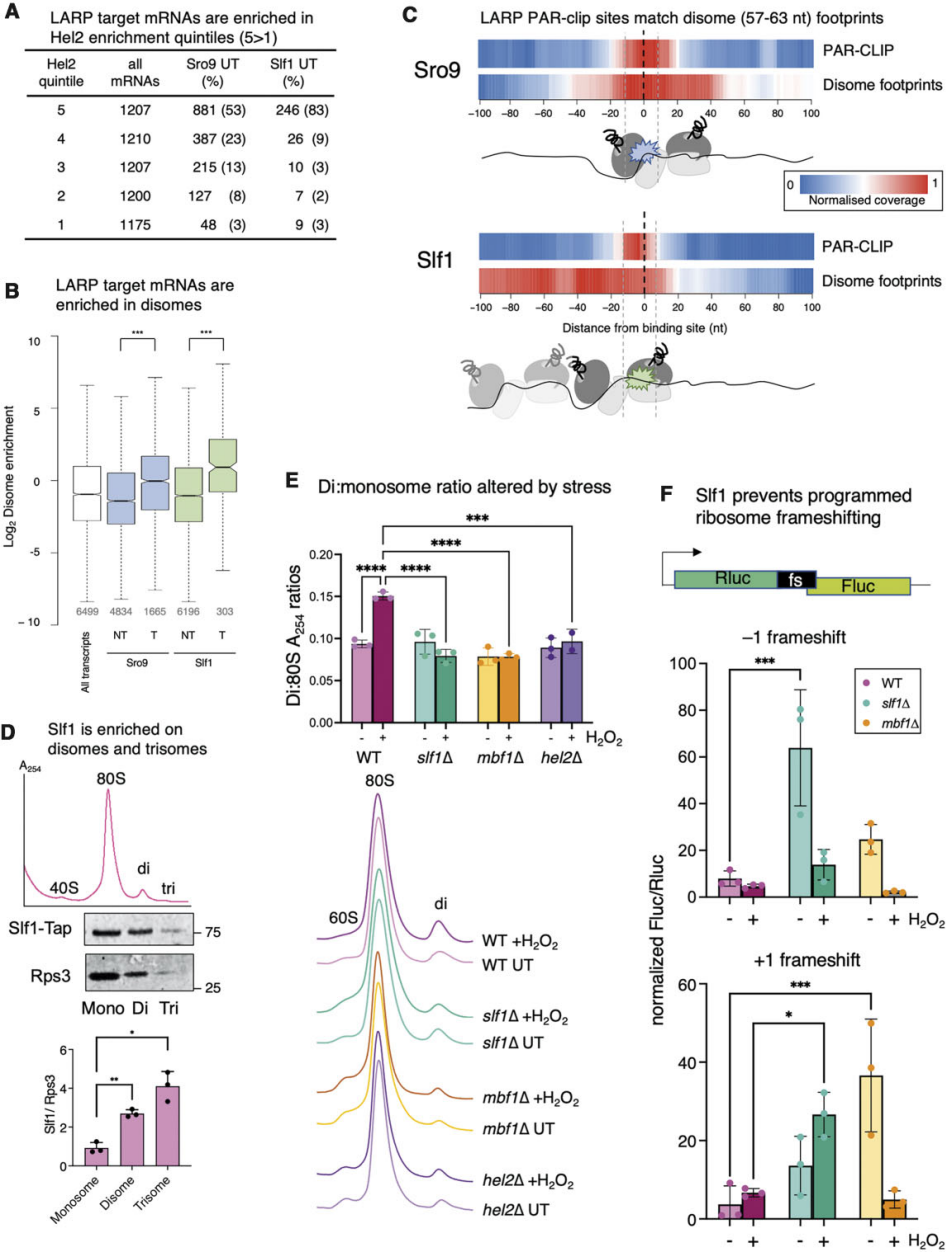
LARPs are enriched on disomes. (**A**) Distribution of Sro9 and Slf1 targets among Hel2 quintiles grouped according to Hel2-CRAC read counts (1, the lowest; 5, highest Hel2 binding), as described (61). (**B**) LARP target mRNAs are enriched in disomes. More disomes are found on Slf1 and Sro9 targets than NT mRNAs. All *P*-values (***) < 2.2 × 10^−16^ (Mann–Whitney test). Box plot details as shown in Figure 1D. (**C**) Meta plot of disomes isolated from untreated cells (40) mapped to ± 100 nt of PAR-CLIP site mode locations. Cartooned enriched disome ribosome and Larp positions shown below (see also Supplementary figure S9). (**D**) Slf1 is enriched on disomes and trisomes. Labelled example sucrose density gradient trace of ribosomes from cell extract not cycloheximide treated (top). Western blots (middle) of collected mono-, di- and trisome fractions, and quantification (*n* = 3). Quantification assumes a single Slf1-binding site per disome/trisome, while two or three Rps3 subunits, respectively are counted. *P*-values two-tailed *t*-test, paired samples ** M:D 0.0015, *M:T 0.017. (**E**) RNase-treated sucrose grandient traces ±15 min peroxide stress, aligned and stacked. Above, quantification of the disome: monosome ratios for 3 biological replicates. Statistics are two-way ANOVA with Tukey post hoc multiple comparison test adjusted *P* values. All pairs indicated **** are < 0.0001, except *hel2Δ* versus WT where *P*= 0.0004. (**F**) DLR assays using programmed + 1 and –1 ribosome frameshifting reporter sequences normalised to frame 0 control values. Cells grown to mid-log ± 2 hour oxidative stress treatment (n = 3). Statistics are two-way ANOVA with Tukey multiple comparison correction among strains; –1 frameshift: *** = 0.0001, +1 frameshift: *** = 0.0004, * = 0.0153.

The shift in relative position of LARPs and 80S ribosomes following stress combined with other observations suggested that the LARPs may help mitigate or resolve ribosome stalls and/or collisions, a role likely more critical during the response to oxidative damage (58). LARP bound mRNAs are abundant with a high density of ribosomes (Supplementary Figure S4), features that may make these mRNAs prone to ribosome collisions. Ribosome stalling can act as a trigger for ribosome associated quality control (RQC) which resolves inactive stalled/collided ribosome pairs, called disomes, and can promote no-go mRNA decay (NGD) of defective transcripts (8,26). One early step in the RQC pathway is thought to be the recruitment of the RING-type E3 ubiquitin ligase Hel2 (ZNF598 in mammals) to ribosomes (59). Both Slf1 and Sro9 are among proteins enriched in Hel2 immune-precipitates (60). Crosslinking and analysis of cDNA (CRAC), analogous to PAR-CLIP, determined Hel2 binding patterns (61). Similar to the yeast LARPs, Hel2 binds ORFs with a 3′ bias, accumulating upstream of stop codons (61). Winz et al. binned yeast mRNAs into five groups (quintiles) by Hel2 binding strength (Hel2-CRAC read counts). We found that the LARP target mRNAs are enriched in the highest Hel2 quintiles (76% of Sro9 and 91% of Slf1 are in the top 40% of Hel2 binding sites, Figure 6A), suggesting a strong correlation between Hel2 and LARP mRNA binding preferences.

As Hel2 binds disomes (62), we asked if the LARP-bound mRNAs are differently enriched with disomes to other RNAs (calculated as disome footprints/mRNA-seq for a disome abundance measure equivalent to TE for monosomes) using mapped disome footprints from unstressed cells (40). LARP-bound transcripts are significantly enriched in disomes over non-target mRNAs (Figure 6B). Metaplots of disome positions shows that these footprints are distributed along ORFs similar to monosome 80S footprints (Supplementary Figure S10A). In addition, and similar to monosome footprints, disomes are enriched at LARP PAR-CLIP binding sites (Figure 6C and Supplementary Figure S10B), implying that the LARPs may bind disomes in addition to 80S monosomes. When the PAR-CLIP, 80S and disome footprints for individual mRNAs were compared the co-alignment patterns of PAR-CLIP and ribosomes are less clear than the metaplots. However, coincident peaks of PAR-CLIP sites with both 80S and disomes are evident (Supplementary Figure S11A-D), particularly for *RPL39* (Supplementary Figure S11D). On these mRNAs disomes are clearly enriched towards the C-terminal half of each ORF which matches the global positioning bias for both LARPs (Figure 2A) and Hel2 (61). Further, disome metaplots appear to show distinct LARP positioning, with a greater enrichment of Sro9 with the rear of the footprint, i.e. the colliding ribosome, while Slf1 enriches at the front of the footprint, i.e. the stalled ribosome region of the footprint plot. These analyses suggest the LARPs act at disomes and hint at possible differences in binding preferences for stalled verses collided ribosomes.

To investigate disome association of Slf1 further, we RNase1-treated cell extracts from *SLF1-TAP* cells followed by fractionation on sucrose gradients to reveal 80S monosomes, disomes and trisomes (Figure 6D). Fractions corresponding to these migration points in the gradients were western blotted. Although Slf1-TAP was found in all ribosome peaks, it was enriched disomes and trisomes compared with monosomes. We next quantified disome:monosome (D:M) ratios from WT and *slf1Δ* cells as well as from cells deleted for known RQC factors Hel2 and Mbf1 (the yeast homolog of mammalian EDF1) (7,63). Loss of Hel2 was found previously to reduce stable disome footprints (40). The D:M ratio increased significantly in WT cells following 15 min H_2_O_2_ treatment (Figure 6E). This is in line with the idea that oxidative stress can modify mRNA (58) and ribosomes (64) leading to slowed elongation (1) and enhanced ribosome stalls and collisions. However, in *slf1*Δ cells as well as *hel2*Δ and *mbf1*Δ cells the D:M ratio was unchanged following stress, suggesting that aspects of the normal stress response were altered following loss of Slf1 or these known RQC factors. Therefore, Slf1 appears to be modestly enriched on disomes and necessary for increased D:M ratios following oxidative stress. These are consistent with the idea that Slf1 can stabilise disomes as previously suggested for Hel2 (40). Cells deleted for Hel2 exhibited high basal levels of eIF2 phosphorylation consistent with the idea that RQC can antagonise activation of Gcn2 kinase (65). We therefore compared eIF2 phosphorylation levels in response to hydrogen peroxide in *slf1Δ*, *hel2*Δ and *mbf1*Δ cells with WT using a phospho-specific antibody. Loss of Slf1 did not alter the observed Gcn2-mediated phosphorylation response to hydrogen peroxide (Supplementary Figure S12A). In contrast *mbf1Δ* cells had a muted response to stress. We conclude that although *slf1Δ* diminishes disome accumulation during peroxide treatment it does not impact Gcn2 activation.

Mbf1 and the ribosomal proteins Asc1 (RACK1) and Rps3 (uS3) were recently implicated in preventing ribosome frameshifting at stalling sequences bearing repeated CGA codons (66). Mbf1 bound the rotated collided 80S near the mRNA entry channel within a disome cryoEM structure (7), suggesting that factors can act with 40S ribosomal proteins to ensure stalled/collided ribosomes maintain reading frame for resumed translation upon stall resolution. Programmed ribosome frameshifting (PRF), common in retroviruses and related elements including retroviral related elements found in yeast, is typically associated with ribosome pauses (67). PRF pauses may be caused by rare codons or by RNA structures within coding regions at the sites of frameshifting. Typically, only a fraction of ribosomes shift reading frame at PRF signals either forward or back and resume translation in the new frame. As Slf1 and Sro9-bound mRNAs have good codon optimality (Supplementary Figure S4E), we addressed if the CLIP sites were enriched in secondary structures. We compared our PAR-CLIP data with a genome-wide secondary structure data set (36). That study derived nucleotide resolution secondary structure scores for over 2600 yeast mRNAs based on single and double-stranded nuclease accessibility. This was termed a parallel analysis of RNA structure (PARS) score, where higher values denote greater structural propensity. We found that Slf1 and Sro9 bound ORFs and 3′UTRs have higher mean (overall per nucleotide) PARS scores than non-targets, with ORFs generally having higher scores than non-coding regions (Supplementary Figure S12B). Next, we calculated the sum PARS score for 30 consecutive bases (PARS30) through each ORF, as 30 nt approximates to a ribosome footprint. Consistent with the mean PARS scores, both LARP target mRNAs have higher mean PARS30 scores than non-target mRNAs (Supplementary Figure S12C). To examine CLIP sites directly we calculated PARS30 scores around the mode location defined mid-point of the top ranked PAR-CLIP binding site in each mRNA. This revealed that the secondary structure propensity was higher at the CLIP-Site (–15 to + 15) than either up- (–45 to –15) or down-stream (+15 to +45)(Supplementary Figure S12C), suggesting that local secondary structure peaks at major PAR-CLIP sites. These analyses suggest that the mapped LARP binding sites may indicate where ribosomes encounter secondary structures. How oxidative stress impacts mRNA secondary structure has not been analysed by similar methods.

To assess if Slf1 can influence PRF, we used two well-characterised DLR systems to monitor +1 and –1 frameshifts from yeast *Ty1* and L–A elements (47). Analogous to the stop-codon readthrough reporters, these PRF reporters place either *Ty1*, L–A or control sequences between out of frame *Renilla* and firefly luciferase ORFs (Figure 6F). WT cells transformed with the DLR plasmids behaved as expected (47) exhibiting 5–10% readthrough that was not altered by hydrogen peroxide (Figure 6F). *mbf1Δ* cells boosted frameshifting of the reporters in unstressed cells, in line with Mbf1′s ability to impact frameshifting at other sequences (66,68) but this effect was eliminated following stress. *slf1Δ* cells also stimulated frameshifting of both PRF reporters: –1 PRF at the L–A element was significantly increased in exponentially growing cells, while stress enhanced +1 frameshifting from the *Ty1* sequence (Figure 6F). While the general reduction in –1 frameshifting seen following stress remains unexplained, it is perhaps paradoxically consistent with the idea that further non-programmed frameshifting could occur during translation of the downstream firefly ORF, thereby reducing functional reporter levels. Taken together these observations suggest that Slf1 may act on ribosomes stalled at regions of secondary structure to help maintain stalled ribosomes in the correct reading frame.

## DISCUSSION

Here we aimed to uncover roles for the yeast LARPs in translation and in the response of cells to oxidative stress. Prior work established that both factors migrate into polysomes and possess both mRNA and 40S association abilities (23–25). Deletion of either protein enhances sensitivity of cells to oxidative stress and RIP-seq showed that the LARPs bound highly translated mRNAs including those encoding antioxidant enzymes (24). Most intriguingly a strain deleted for Slf1 failed to promote expression of antioxidant proteins, likely explaining the enhanced oxidant cellular toxicity (24). These observations pointed to a role for the yeast LARPs in translational control but did not suggest a specific mechanism. Here, we performed PAR-CLIP, and found that both factors selectively bind subsets of highly translated mRNAs, primarily via binding within ORFs. Slf1 bound a subset of Sro9 mRNAs, but not necessarily at the same positions within each ORF. The global trend was few LARP binding sites near the AUG, but a steady increase through the ORF to the stop codon. Stress had only a modest impact on both the identity of target transcripts and the binding positions within each ORF. Further experiments with *slf1*Δ cells indicate it impacts production of antioxidant proteins during stress without affecting the transcriptional induction of antioxidant mRNAs, their interaction with the mRNA cap-binding factor eIF4E or via preventing antioxidant mRNA engagement with 80S ribosomes. *slf1*Δ did not impact rates of stop-codon readthrough on reporter transcripts. Thus, it appears that translation is impacted by loss of Slf1 during translation elongation itself. The framing of each end of the LARP CLIP protected fragments relative to ORF codons as well as the remarkable enrichment of ribosome footprints at the LARP mRNA binding sites, as viewed in metaplots for both 80S and disome footprints, strongly suggest that the LARPs are engaged primarily with elongating and/or stalled ribosomes.

One observation from the footprint metaplots is that during stress the positions of ribosome footprints relative to the LARP PAR-CLIP sites change and show both LARP-specific and ribosome footprint size distinct patterns. In stressed cell samples both LARP cross-linking positions are more enriched in the spaces between 80S ribosome peaks. A distinction between LARPs is Sro9 has more 80S footprints ahead of the protein CLIP sites, including enrichment for shorter 21–22 nt 80S footprints that are indicative of rotated 80S with empty A sites in a pre-accommodation state, presumably waiting for an incoming tRNA (69). In contrast the 21–22 nt 80S footprints accumulate behind Slf1 CLIP positions during stress (69). Positioning the LARPs between 80S footprints may help limit ribosome collisions during stress. Disome footprinting has not been reported for peroxide stressed cells, but in unstressed cells disomes accumulate at the CLIP sites for both LARPs. The relative position of each LARP/disome plot is indicative of preferential binding of Slf1 to the stalled ribosome and Sro9 to the rear collided ribosome. Slf1 footprints have fewer 80S and disome footprints ribosomes ahead of them than Sro9 does, suggesting Slf1 is binding to a leading ribosome on any given mRNA. This may be attributed in part to the proximity of stop codons downstream. We found that Slf1 associates with disomes and that loss of Slf1 does impact disome accumulation during stress, similar to the loss of known disome-associated factors Hel2 and Mbf1. However, *slf1*Δ cells did not alter the Gcn2 mediated ISR phosphorylation of eIF2. Consistent with roles for the LARPs in resolving ribosome stalls, *slf1*Δ and *sro9*Δ cells display altered sensitivity to the antibiotic paromomycin which reduces translation elongation fidelity and can promote –1 ribosomal frameshifting (23). Mbf1 mutants impact + 1 ribosome frameshifting at RQC promoting CGA repeat stalling sequences (66) and at other yeast mRNAs (68) including *LEU2, MET2 and HIS4*. Curiously some frameshift sites identified previously in *LEU2* and *HIS4* contain GGU codons, similar to the motif enriched in our LARP PAR-CLIP experiments. The L–A viral –1 frameshift promoting sequence we used also possesses a GGU codon in the 0 frame of the shifting element (47). *slf1Δ* significantly boosted frameshifting of the L–A DLR, while *mbf1*Δ promoted + 1 frameshifting of the *Ty1* DLR. Recently ISR regulators Gcn1 and Gcn20 which bind disomes (7) were also found to antagonise frameshifting at CGA repeat stalling sequences, while in contrast the fungal-specific elongation factor eEF3 promoted frameshifting (70).

Our results suggest that Slf1 acts as one of several factors that can bind stalled and collided ribosomes. Slf1 specifically interacts with a subset of endogenous mRNAs that are among the most translationally active mRNAs and are hence engaged with a high density of ribosomes. The translation of antioxidant mRNAs is critical for cells to adapt to cellular stress. We propose that Slf1 helps maintain ribosomes in the correct reading frame. When deleted, enhanced frameshifting at multiple points along these mRNAs could lead to aberrant expression of truncated protein forms that are likely degraded. This could account for the observations that in *slf1Δ* cells ribosomes remain engaged with antioxidant and other mRNAs during stress, but that protein levels stay low.

Precisely how Slf1 could achieve this remains unclear. The apparent shift in the position of Slf1 to behind between 80S ribosomes during stress may indicate it changes from being mainly a 40S binding factor to being mRNA-bound. The Slf1 La related domain is needed for mRNA binding (25) while its amino terminal region promotes 40S ribosome interactions (24). Perhaps Slf1 could act like a brake or clamp to help prevent ribosome frameshifts. It may act as a buffer to stop the next ribosome colliding with the stalled ribosome. Secondly when ribosomes do collide Slf1 binding appears to stabilise disomes, which may facilitate resumed translation of mRNAs needed for stress resolution and a return to growth. Our CLIP/disome footprint meta-analyses point towards a model where Slf1 favors binding to stalled ribosomes, while Sro9 is more enriched at collided ribosomes. Delineating their precise roles will require further study.

More broadly, how do these findings relate to others LARPs? In humans LARP1 binding to mRNAs has been assessed by PAR-CLIP (18). However the binding of LARP1 is complicated because it additionally possesses a DM15 domain that promotes binding to ribosomal protein and translation factor mRNAs bearing 5′ terminal oligo-purine tracts (5′TOP) at their 5′ ends (71). 5′ UTR/ 5′TOP binding accounted for ∼5% of LARP1 binding to ribosomal protein mRNAs, while ∼60% of the binding was within the ORFs of these mRNAs. ORF binding was higher for non-5′TOP transcripts (18). A metaplot of ORF binding revealed a pattern of PAR-CLIP reads accumulating through the ORF and peaking just before the stop codon (18), similar to the patterns we describe for Slf1 and Sro9. Although much of the research focus for LARP1 biology in recent years has centered on its 5′TOP mediated control, these observations suggest that LARP1 may play multiple roles including via ORF or ribosome-binding. Although much remains to be uncovered, the findings we report here for the yeast LARP proteins may point to a conserved core LARP role that is retained in human cells.

## DATA AVAILABILITY

Sequencing files are deposited with the GEO accession number GSE174707. All other resources generated are available from the corresponding author.

## Supplementary Material

gkad272_Supplemental_Files

## Notes

Present address: Martin D. Jennings, Peak Proteins Birchwood House, Larkwood Way, Macclesfield, Cheshire, SK10 2XR, UK.

Present address: Christopher J. Kershaw, North West Genomics Laboratory Hub (Manchester), Manchester Centre for Genomic Medicine, St. Mary's Hospital, Oxford Road, Manchester, M13 9WL, UK.
