# Supplementary material for: Interaction of the La-related protein Slf1 with colliding ribosomes maintains translation of oxidative-stress responsive mRNAs
Source: Nucleic Acids Res. 2023 Apr 18;51(11):5755–73. doi: 10.1093/nar/gkad272 (PMC10287931; doi:10.1093/nar/gkad272)
Supplement: gkad272_Supplemental_Files [file gkad272_supplemental_files.zip › Supplementary Materials_revision2.pdf]

Supplementary Materials for

Interaction of the La-related protein Slf1 with colliding ribosomes maintains translation of oxidative-stress responsive mRNAs.

Martin D. Jennings[1,2,5], Priya Srivastava[1,5], Christopher J. Kershaw[1,3], David Talavera[4], Christopher M. Grant[1] and Graham D. Pavitt[1,*]

The supplementary materials included in this file are
Supplementary Figures 1-12,
source uncropped western blot images and
supplementary references.

In addition, Supplementary Tables1-10 are found in two separate multi-tab excel spreadsheet files:

Supplementary Tables 1-6.xlsx -contains data relating to the main and supplementary figures.

Supplementary Tables 7-10.xlsx -contains methods related details.

Supplementary Table 1. Total mRNA-seq read numbers from LARP PAR-CLIP experiments
Supplementary Table 2. Sequencing and processing numbers from PAR-CLIP experiments
Supplementary Table 3. Per gene RNAseq and PAR-CLIP analyses
Supplementary Table 4. Read numbers from mRNA-seq of WT, *sro9*Δ and *slf1*Δ strains without and with H_2O_2 treatment
Supplementary Table 5. DESeq analysis of peroxide induced transcript changes WT, *sro9*Δ and *slf1*Δ strains without and with H_2O_2 treatment**.**
Supplementary Table 6. DESeq analysis of P:M enrichment of mRNAs in WT and *slf1*Δ cells.
Supplementary Table 7. Yeast strains used in this study
Supplementary Table 8. Plasmids used in this study
Supplementary Table 9. Oligonucleotides used in this study
Supplementary Table 10. Key Resources Used.

Supplementary Figures

Supplementary Figure 1. Sro9 and Slf1 homology, stress response and CLIP complexes

(related to Figure 1)

A Similarity alignment of the yeast LARPs using MUSCLE (1). LaM and previously determined region necessary for Slf1-ribosome binding (2) are shown. Sites of interaction with the ribosomal protein Asc1 (3) or crosslinked (XL) to RNA (4) are indicated.

B eIF2 phosphorylation in response to H_2O_2 stress maintained in TAP tagged strains used for CLIP. Immunoblot and total protein stain for indicated strains ± 0.8 mM Hydrogen peroxide for 15 minutes. Right: Quantified signals normalized to total protein loaded across blot to mean signal ±SD (n=3). Statistics: 2-way ANOVA with a Tukey *post hoc* test. *P* values: WT =0.0009, Slf1 tap= 0.0002, Sro9 tap= 0.0001.

C ^{32}P RNP-complexes UV crosslinked (+) or not (− control) resolved on a 4-12% NU-PAGE bis-tris gel. * indicate RNA-protein complexes formed.

Supplementary Fig. 1

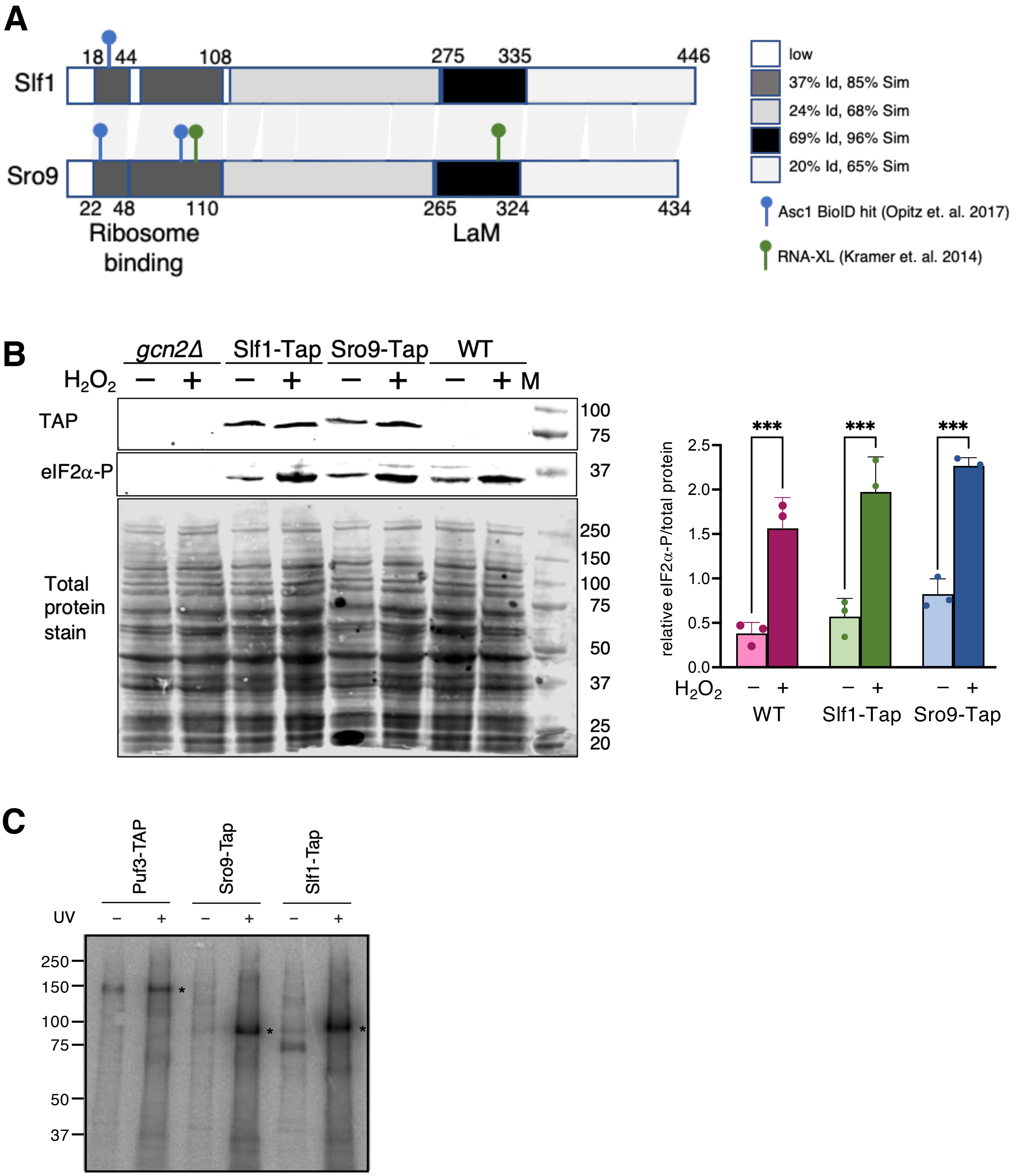

Supplementary Figure 2. Puf3 PAR-CLIP analysis
(related to Figure 1)
A Table of PAR-CLIP reads and processing for Puf3.
B Annotation of Puf3 binding sites.
C Aggregate mRNA binding site distribution relative to start codons (left), stop codon (right) and across the ORF (middle) for Puf3.
D Sequencing coverage on *COX17* showing mapped read numbers for PAR-CLIP from Puf3.
E The most enriched motif identified from Puf3 PAR-CLIP identified using MEME.

Supplementary Fig. 2

A

	Puf3
Raw reads (M)	5.6
Trimmed and filtered reads (M)	3.0
Mapped reads (M)	1.5
Total binding sites	2148
mRNA binding sites	1809
Total targets	1474
mRNA targets	1225

B

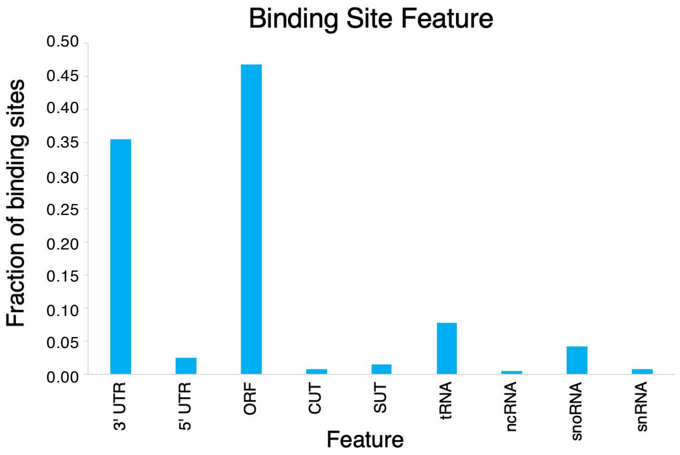

C

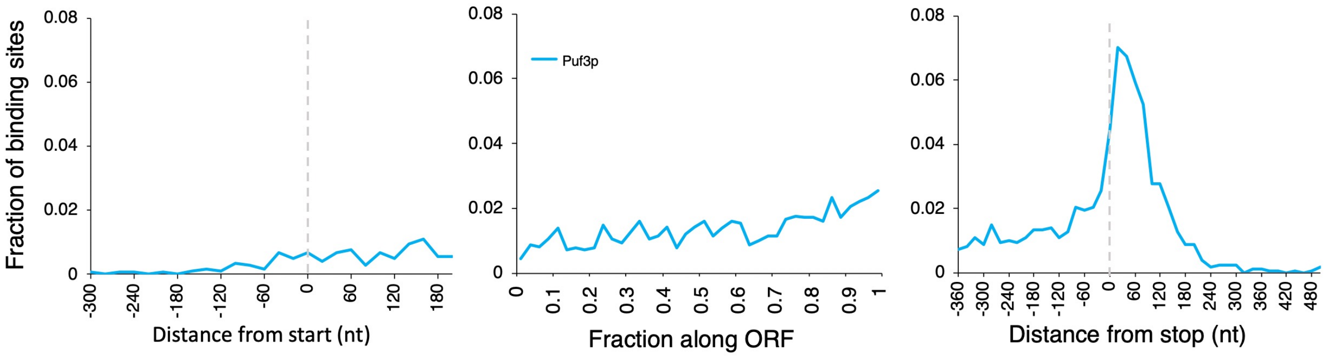

D

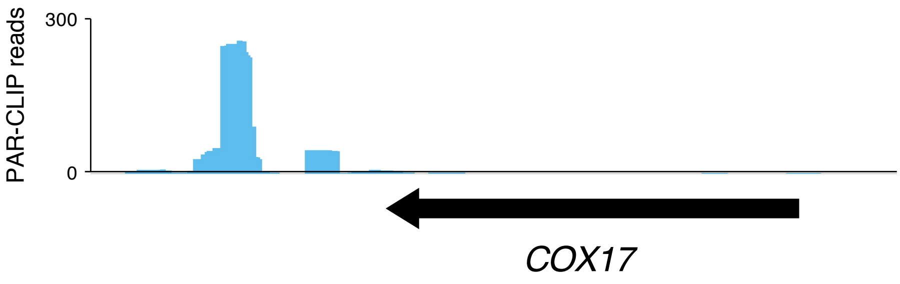

E

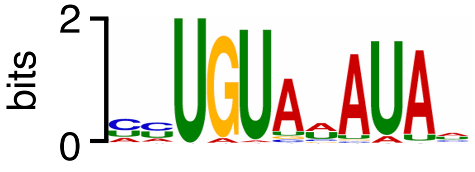

Supplementary Figure 3. Global comparison of Sro9 and Slf1 PAR-CLIP target sites

(related to Figure 1)

A Total number of 3'UTR, ORF and 5'UTR binding sites within individual mRNAs across all targets. Boxes extend from 25-75% of the data range with notches around median. The notches are +/-1.58* interquartile range(IQR)/sqrt(n) and represent the 95% confidence interval for each median. Whiskers extend to data points that are less than 1.5 x IQR away from 1st/3rd quartile. The number of mRNAs in each group is given below each plot.

B 3'UTR ± 5'UTR sites are mainly in mRNAs where ORF is also bound. Venn style diagrams of binding site overlaps.

C Sro9-TAP and Slf1-TAP protein levels. Quantification shown relative to Slf1. Sro9-TAP mean value = 13.5. Error bars represent s.e.m (n = 6)

D, E Comparisons between PAR-CLIP targets (from this study) and RIP-Seq targets identified in Kershaw et al 2015, in either H_2O_2 treated (T) or untreated (UT) cells. **D** Sro9-TAP. **E** Slf1-TAP.

Supplementary Fig. 3

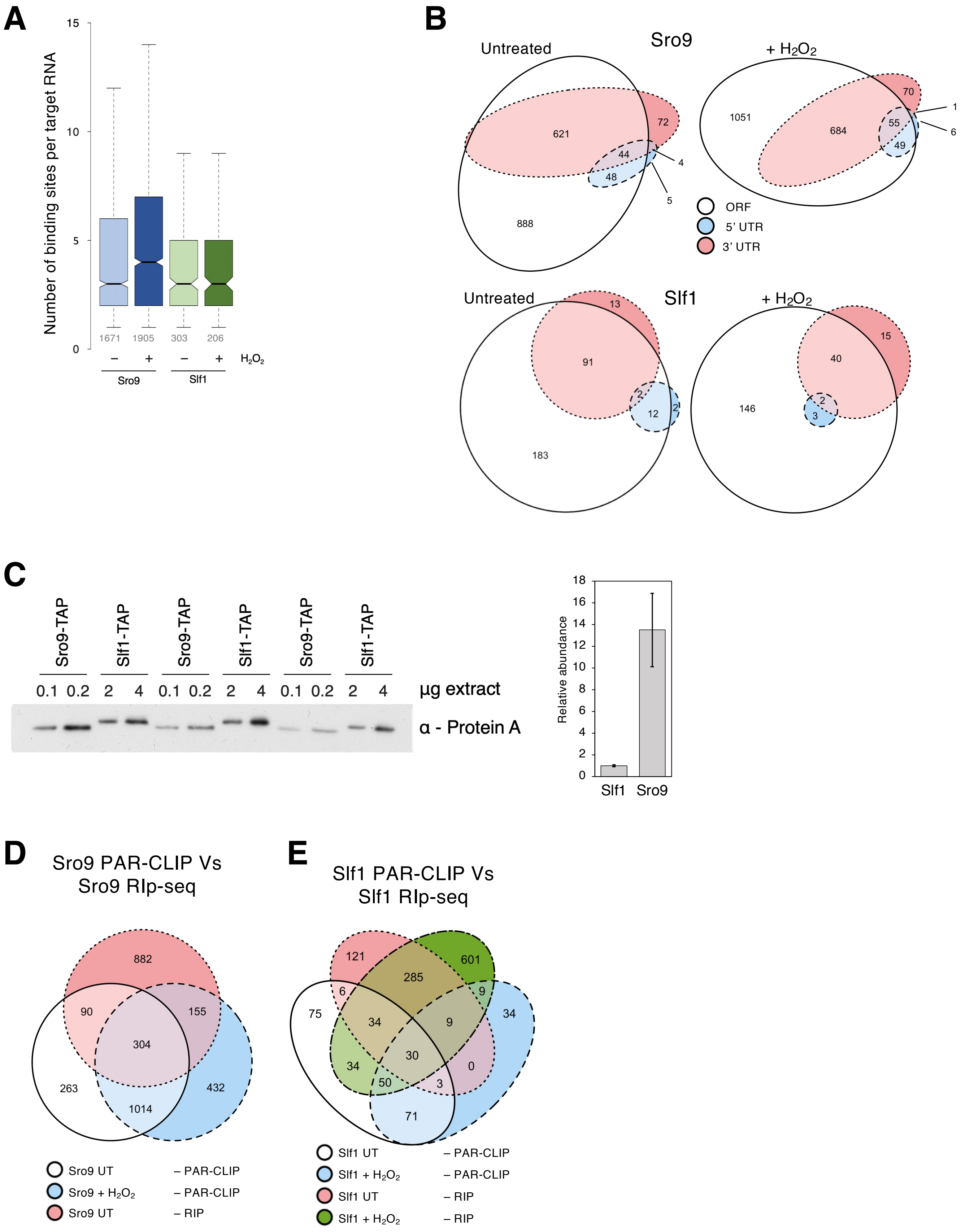

Supplementary Figure 4. Slf1 and Sro9 targets are abundant and efficiently translated.

(related to Figure 1)

Features of all yeast protein coding transcripts (white/grey) or those targeted (T) or not targeted (NT) by Slf1 (green) or Sro9 (blue) both in the absence (−) or presence (+) of H_2O_2 shown in Box and whisker plots. Boxes extend from 25-75% of the data range with notches around median. The notches are +/-1.58* interquartile range(IQR)/sqrt(n) and represent the 95% confidence interval for each median. Whiskers extend to data points that are less than 1.5 x IQR away from 1st/3rd quartile. The number of mRNAs in each group is given below each plot. *P*-values were calculated using the Mann-Whitney test.

A 5' UTR length in bp (*p*-values left to right: ** 0.001117, * 0.01317, *** 7.715×10^{-6}, *** 8.677×10^{-6}).

B ORF length in bp. (p-values left to right: * 0.03358, *** 3.067×10^{-09},*** 4.696×10^{-05}, *** 1.001×10^{-05}).

C 3' UTR length, all are non-significant (ns).

D Calculated transcript abundance in FPKM in untagged strains, from this study (*p*-values *** all < 2.2×10^{-16}).

E Average tRNA adaptation index (tAI)(5) across each ORF (p-values *** all < 2.2×10^{-16}).

F Log_2 Ribosome density values (ribosomal rpkm/mRNA rpkm) in unstressed cells calculated from(6) and these data (*p*-values left to right *** < 2.2×10^{-16}, < 2.2×10^{-16}, < 2.2×10^{-16}, 2.68×10^{-12}). The sum of both 28-29 and 21-22 nt footprints was used(6).

G Translation initiation efficiency(7) (*p*-values *** all < 2.2×10^{-16}).

Supplementary Fig. 4

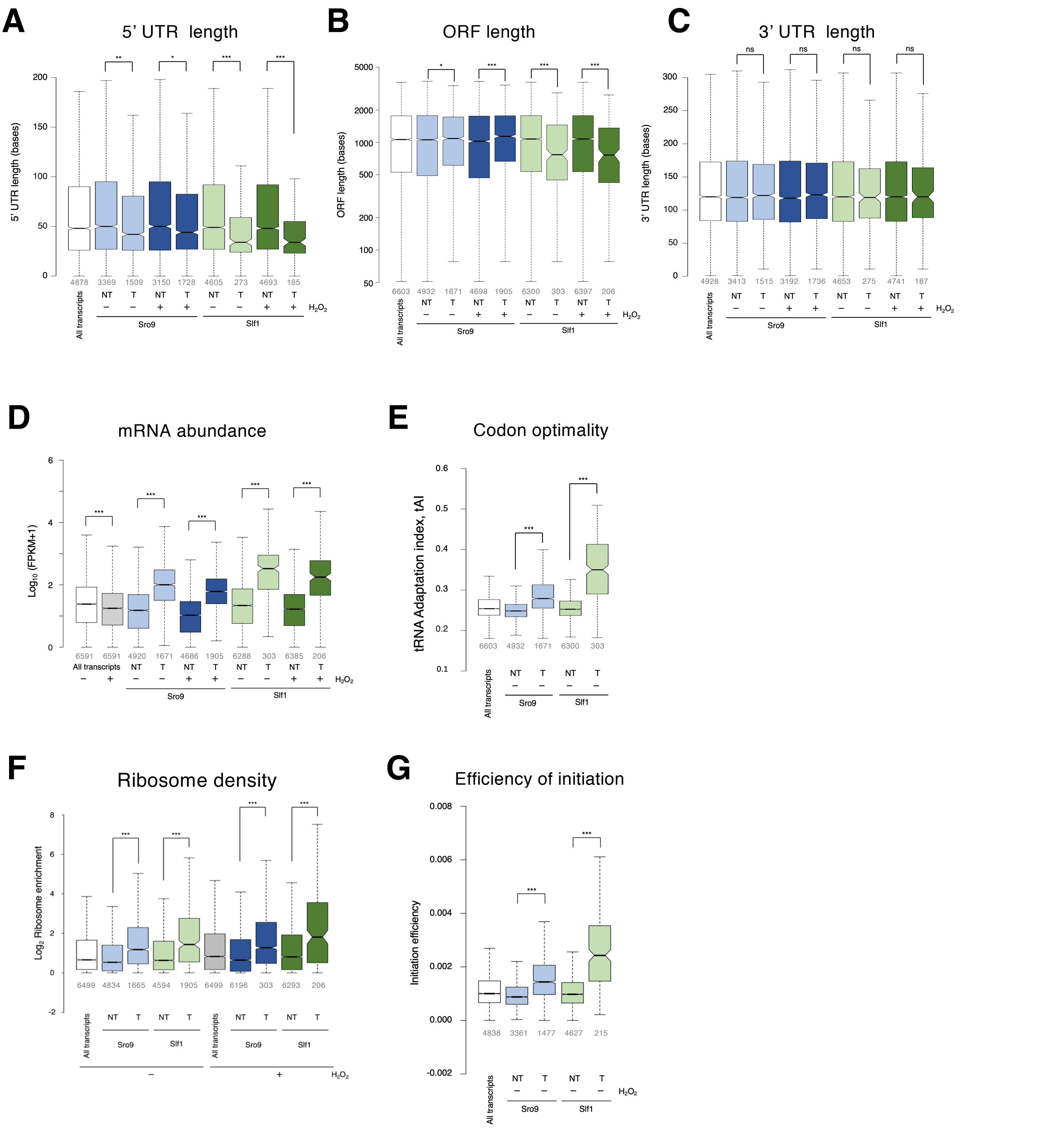

Supplementary Figure 5. PARCLIP reveals LARP-mRNA interactions increase through the ORF

(related to Figure 2)

A Top: Histograms showing normalized sequence read coverage across ORFs from PAR-CLIP experiment. Left, read coverage across transcripts from the total RNA samples. Right, reads identified from PAR-CLIP of Sro9 or Slf1 in untreated (UT) or peroxide treated conditions (+ H_2O_2). Transcripts are split into 100 quantiles.

Bottom: left 80S monosome ribosome footprint read from reference (6). Ribosome reads are from either WT (- H_2O_2) or stressed (+ H_2O_2) conditions for all mRNAs or LARP target mRNAs from each condition. Right, Key to histograms.

B Sequencing coverage from PAR-CLIP (colored) or mRNA-seq (grey) from H_2O_2 treated or untreated (UT) yeast. *TSA2* (top) and *TRX2* (bottom). Each ORF is shown by a horizontal black arrow from AUG to stop (arrowhead).

Supplementary Fig. 5

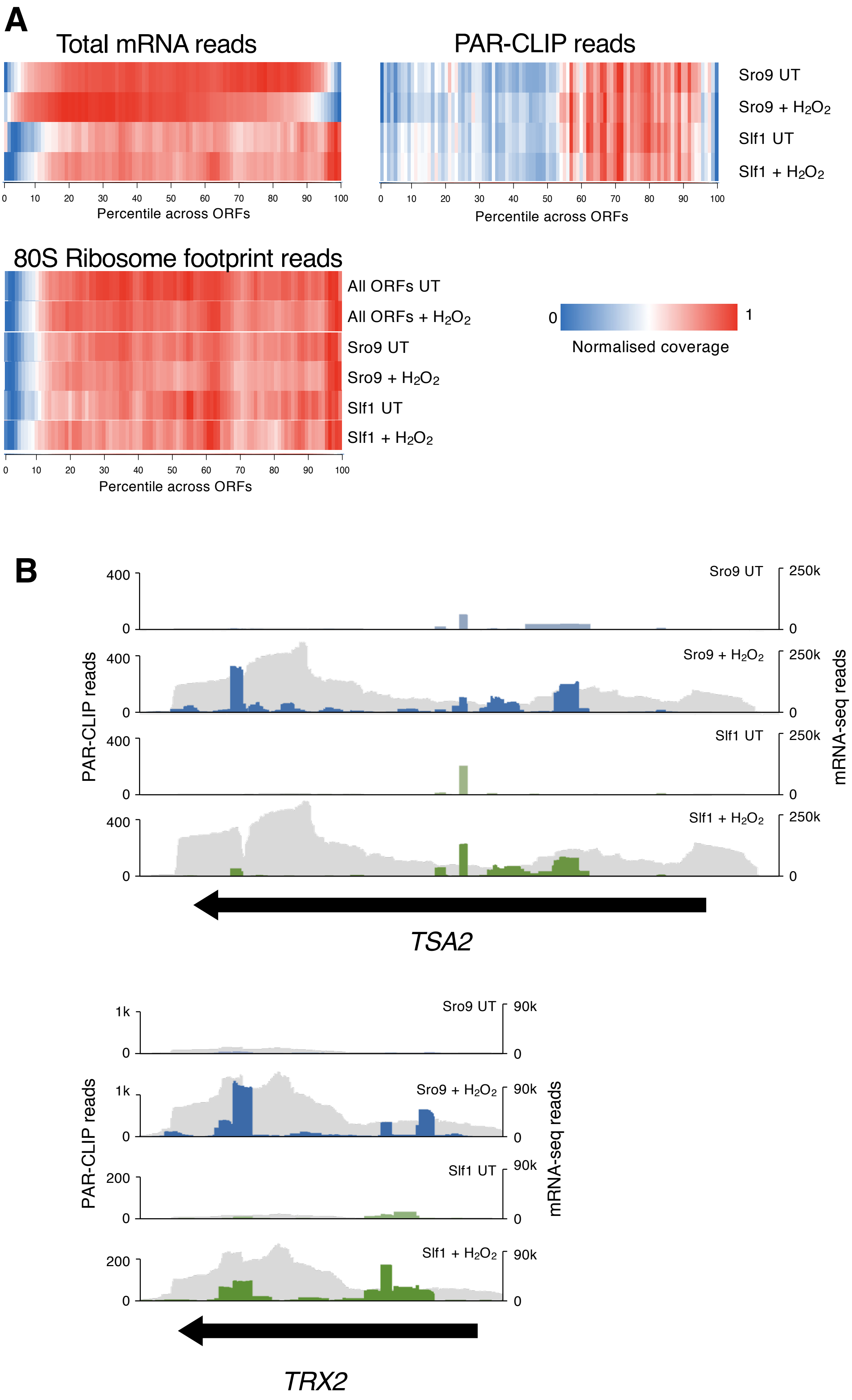

Supplementary Figure 6. The oxidative stress transcriptional response is maintained in LARP mutant cells.

(related to Figure 3)

A qRT-PCR of transcript levels relative to a luciferase spike-in control. n=3. error bar =SD.

B Measurement of β-galactosidase activity from a YRE-LacZ reporter in Wild-Type (WT), *sro9*Δ and *slf1*Δ strains either in the absence (−) or presence (+) of H_2O_2.

C Gene ontology (GO) of mRNAs significantly upregulated by peroxide in WT and LARP deleted cells (top) and comparison of the $\log_2$ fold change (FC) of YAP1 response target genes in WT and *slf1* strains upon H_2O_2 treatment, as calculated with DESeq2.

D Western blot detection of oxidative stress response proteins either in the absence (−) or presence (+) of H_2O_2. Protein levels in WT, *sro9*Δ and *slf1*Δ strains, Pgk1 is a non-antioxidant loading control. Quantification of bands is shown below panels - relative to the first lane and normalized to Pgk1.

Supplementary Fig. 6

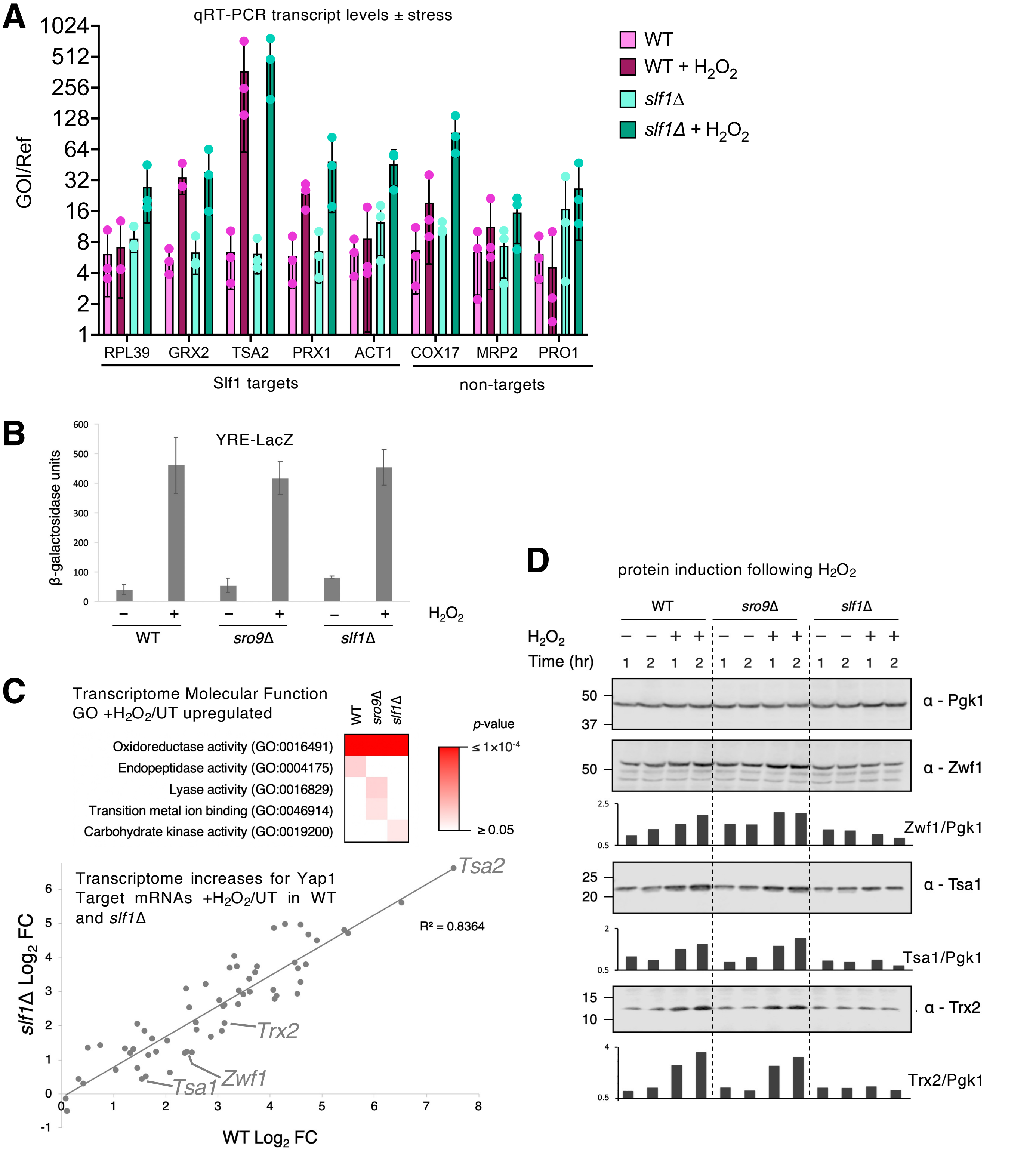

Supplementary Figure 7. Stop codon readthrough and eIF4F enrichment with LARP targets
(related to Figure 3)
A Dual luciferase reporter assays for stop codon readthrough show stress and *slf1*Δ do not impact recognition of each of the three stop codons (n=3).
B LARP-bound mRNAs are enriched in eIF4F. Significantly (FDR < 0.05) positively enriched (red, fold enrichment >0) or negatively enriched (blue, fold enrichment <0) transcript groups from Costello et al 2015(8) binding to Sro9 or Slf1 from H_2O_2 treated or untreated (UT) yeast. *P*-value coloring is from a Fisher's Exact test.
C Control Input and supernatant probing of eIF4E-TAP with anti-PAP antibodies. Almost all eIF4E is depleted from the supernatant.

Supplementary Fig. 7

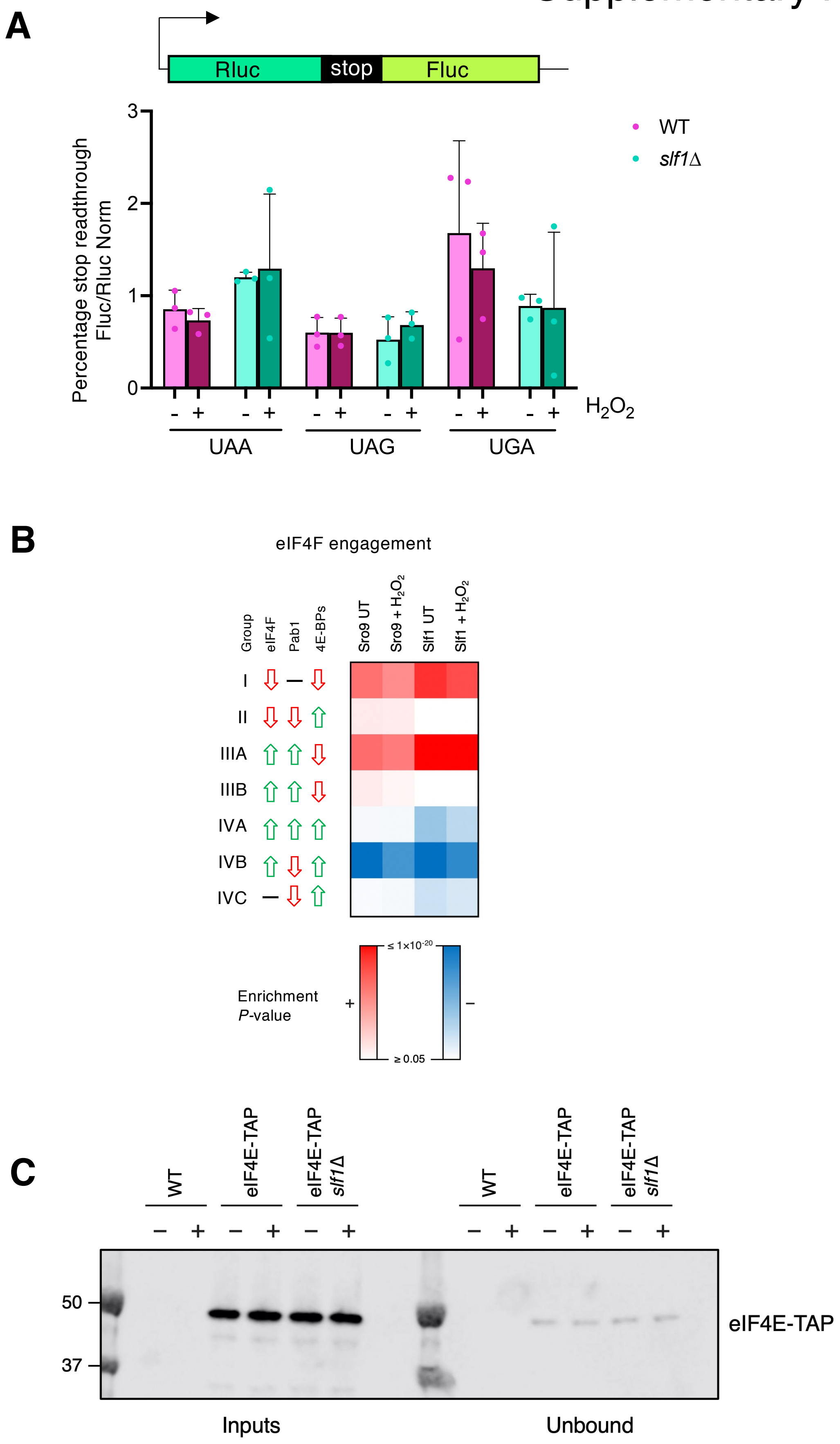

Supplementary Figure 8. YGSU motif frequency and framing.
(related to Figure 5)
A Proportion of Sro9 and Slf1 PAR-CLIP binding sites from H_2O_2 treated or untreated (UT) yeast containing different numbers of YGSU motifs.
B Fraction of ORF binding site start and end points in each of the three possible reading frames. Cartoon below gives theoretical example.
Chi square test adjusted *P*-values indicate both 5' ($4.35x10^{-114}$) and 3' ($3.56x10^{-141}$) patterns are non-random.
At the 5' end there is overrepresentation for frames 0 (P = $6.66x10^{-11}$) and +1 (P = $3.25x10^{-54}$), and depletion for frame +2 (P = $2.41x10^{-109}$).
At the 3' there is overrepresentation for frames 0 (P = $6.61x10^{-43}$) and +2 (P = $1.01x10^{-30}$), and depletion for frame +1 (P = $3.73x10^{-142}$).
C YGSU containing binding site 5' and 3' end framing is independent of binding site length. Plots shows nucleotide distance from first YGSU to binding site 5' end and from last YGSU to the 3' end. Frame bars colored as in B.

Supplementary Fig. 8

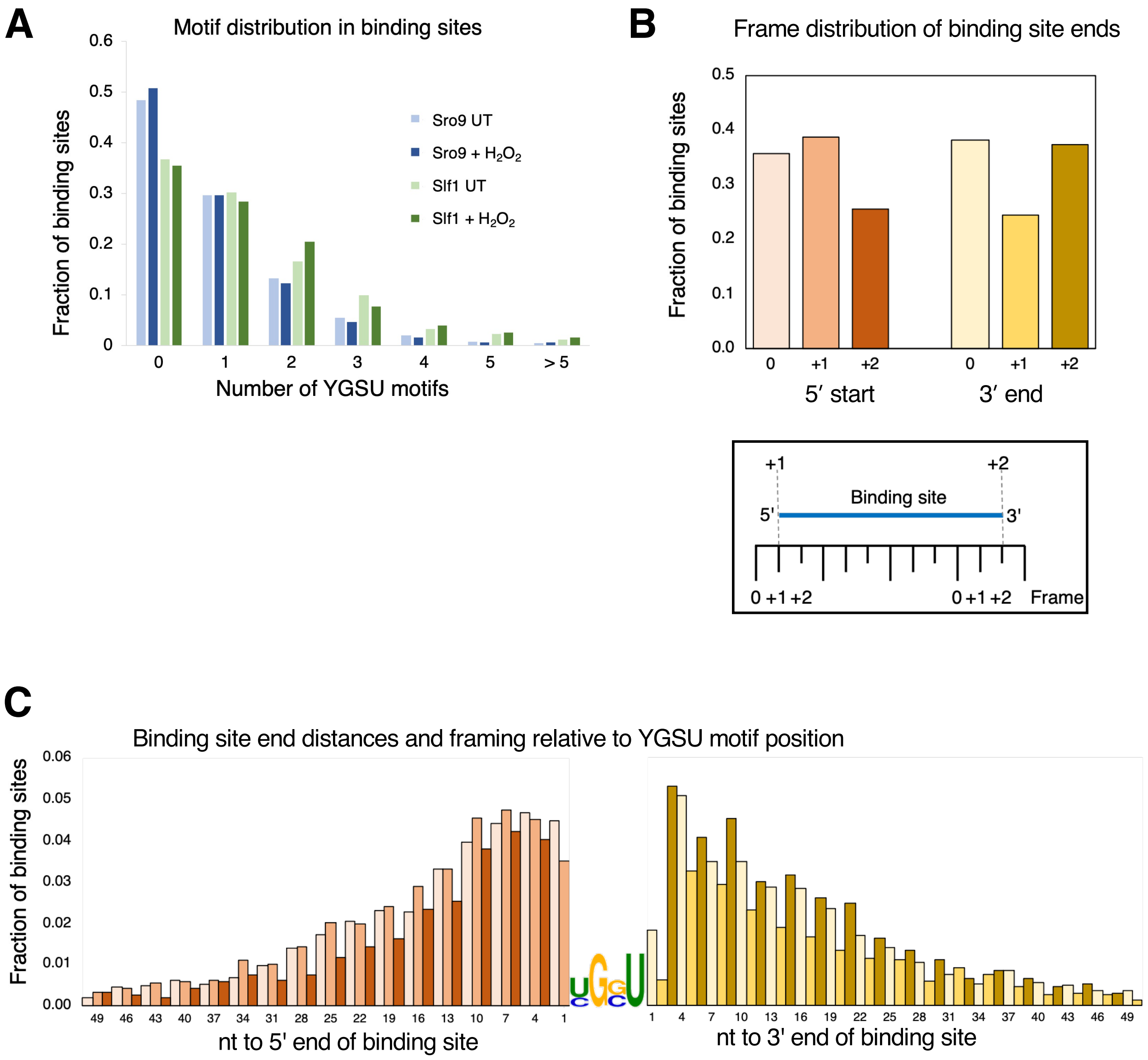

Supplementary Figure 9. tRNA copy number and enrichment in PAR-CLIP reads (related to Figure 5)
A tRNA copy number in *S. cerevisiae*.
B tRNA read counts across all 4 PAR-CLIP data sets. GGU and GCU decoding tRNAs highlighted with arrows.

Supplementary Fig. 9

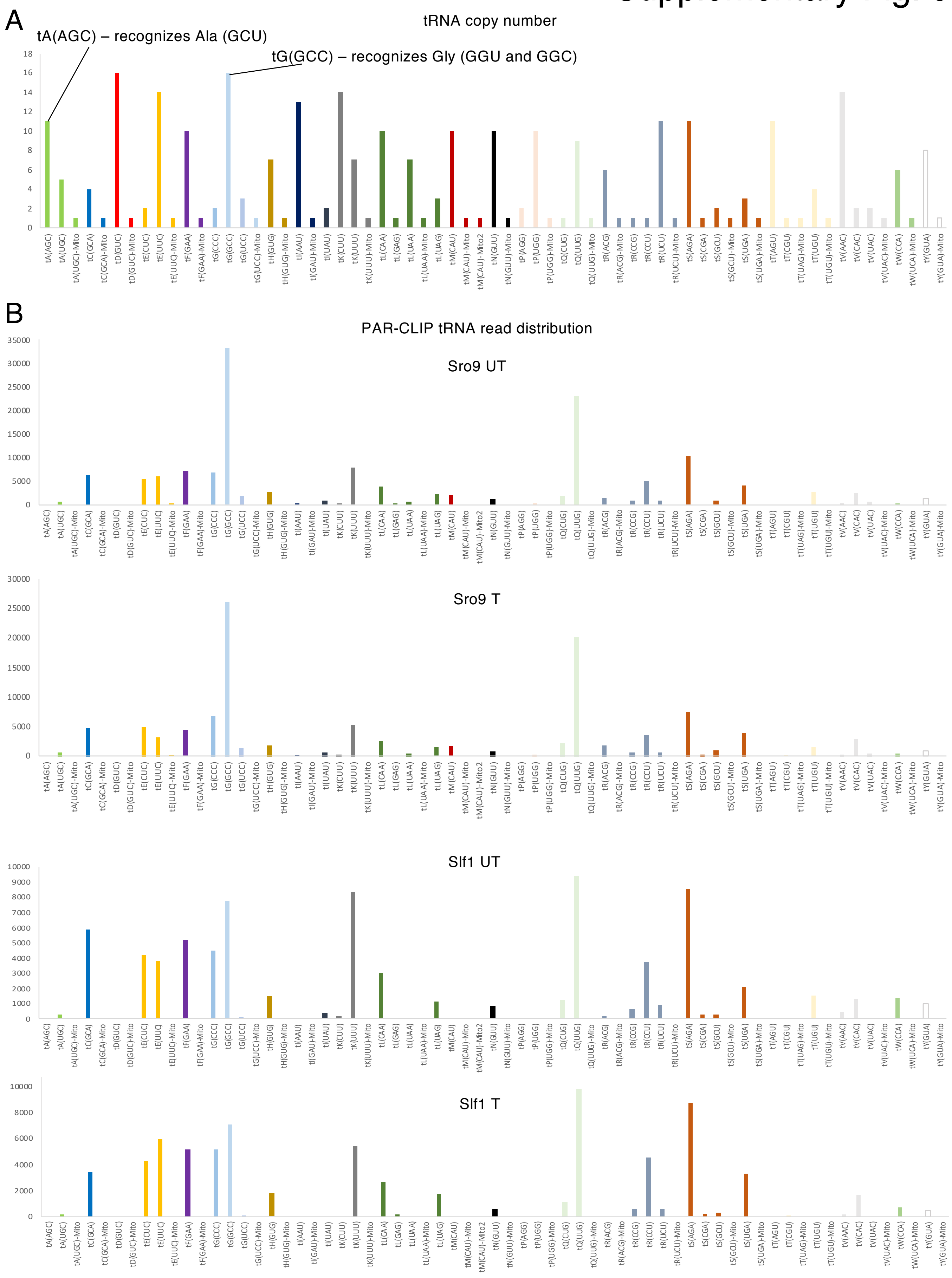

Supplementary Figure 10. Ribosome footprints are enriched at LARP PAR-CLIP sites
(related to Figure 5)
A Ribosome footprint read coverage across all ORFs or PAR-CLIP target ORFs only
B Ribosome footprinting read coverage around PAR-CLIP binding sites (centered at 0) with increasing window sizes (+/- 100, 200, 300 and 500 nts).
Histograms show normalised ribosome read coverage as indicated in the key. Ribosome reads are from either WT (- H_2O_2) or stressed (+ H_2O_2) conditions.
80S monosome reads are from(6) and represent either all ribosome reads or those from standard footprints (28-29 nts) or small footprints (21-22 nts) only. Disome footprints (57-63 nt) are from Meydan and Guydosh(9). Transcripts/regions were split into 100 quantiles.

Supplementary Fig. 10

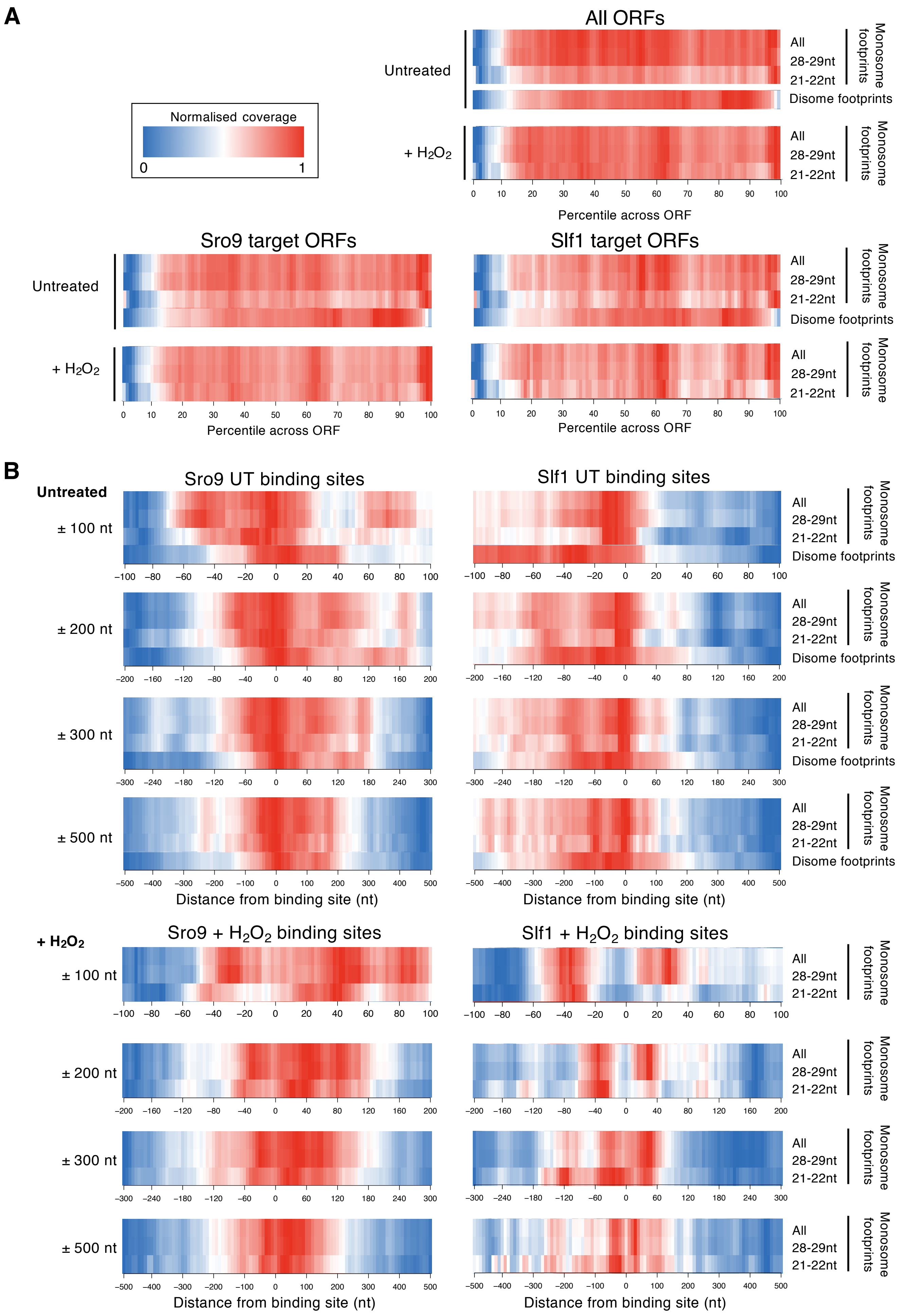

Supplementary Figure 11. Alignment of PAR-CLIP, RNAseq, monosome and disome sequencing reads for four genes.
(related to Figure 6).
A-D Plots of mapped RNA-seq reads from PAR-CLIP and RNA-seq (this study, as shown in Figure 2) with monosome(6) and disome(9) footprints for the named yeast genes. Vertical black dotted lines provide a visual aid to two major PAR-CLIP binding positions in each gene. Each ORF is shown by a horizontal black arrow showing its direction of translation. The intron in *RPL39* is shown as a dashed line.

Supplementary Fig. 11

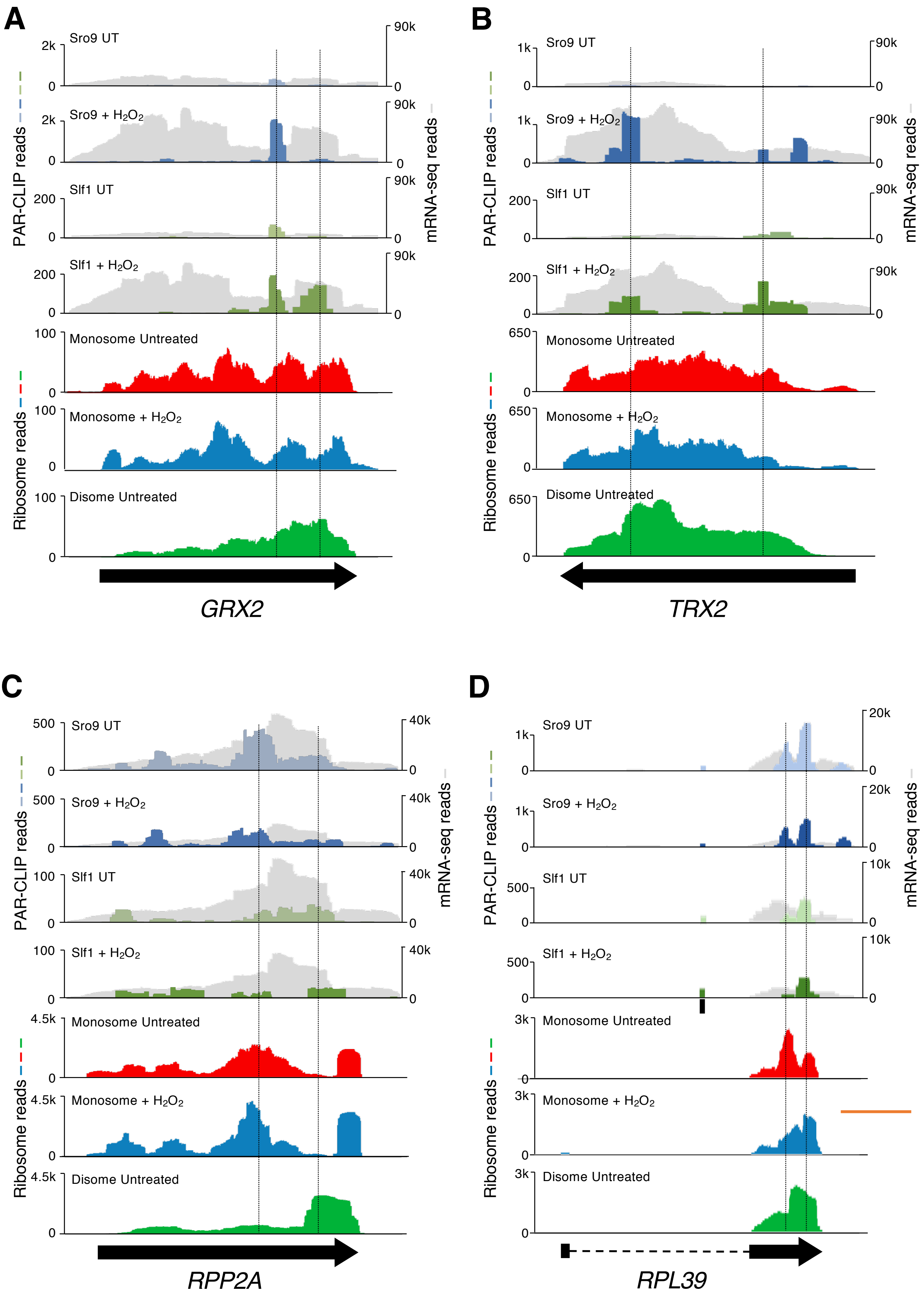

Supplementary Figure 12. eIF2 phosphorylation and PAR-CLIP target RNA secondary structures.

(related to Figure 6).

A *slf1*Δ does not impact eIF2αP ISR response. Immunoblotting with phospho-specific antibody to eIF2 alphaStatistics show two-way ANOVA ****<0.0001

B and **C** Box and whisker plots of PARS secondary structure analysis applied to PAR-CLIP datasets. Boxes extend from 25-75% of the data range with notches around median. The notches are +/-1.58* interquartile range(IQR)/sqrt(n) and represent the 95% confidence interval for each median. Whiskers extend to data points that are less than 1.5 x IQR away from 1st/3rd quartile. The number of mRNAs in each group is given below each plot. **B** Mean PARS scores per base for 5'UTRs, ORF and 3'UTRs for target (T) and non(T) Sro9 (blue) and Slf1 (green)and all gene controls. *P*-values were calculated using the Mann-Whitney test **** <0.0001. **C Left** mean PARS30 (sum of PARS scores for 30 consecutive bases) across entire mRNAs (groups defined as in B) or **right** PARS30 around major PAR-CLIP mode locations only (as defined as the best CLIP site in each target gene by Paralyser analysis of PAR-CLIP features). Inset cartoons show PARS30 positions relative to PAR-CLIP sites. *P*-values were calculated using the Mann-Whitney test **** <0.0001, Sro9 clip sites: ***p=0.0002 **p=0.0013, Slf1 clip sites ** p=0.0061

Supplementary Fig. 12

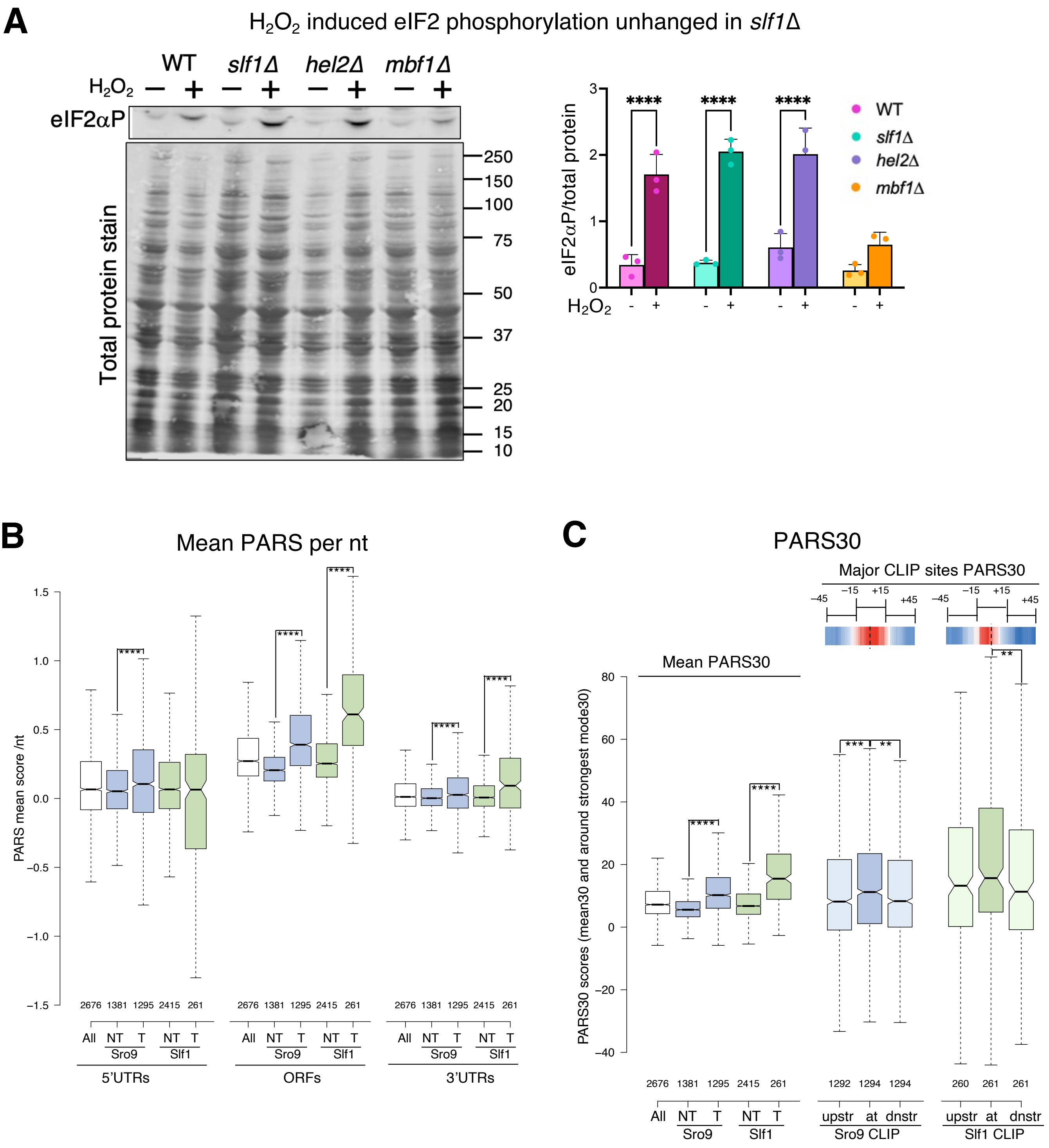

Source data.

Uncropped western blot images relating to Figures 3A, 6D and Supplementary Figures 1B, 1C, 3C, 6D, 7C, 12A

source western blots for figures and supplementary figures

Fig. 3A

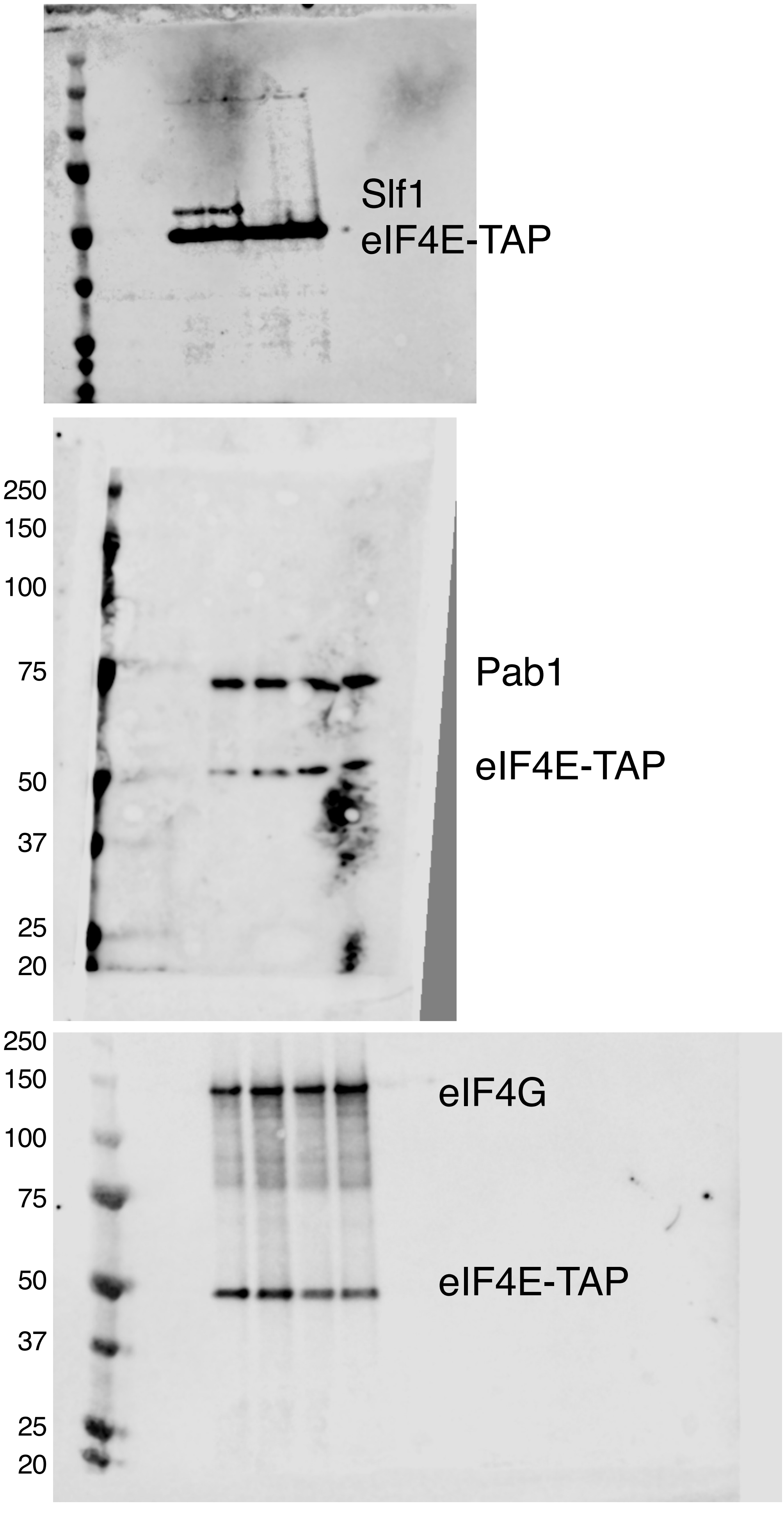

Fig. 6D

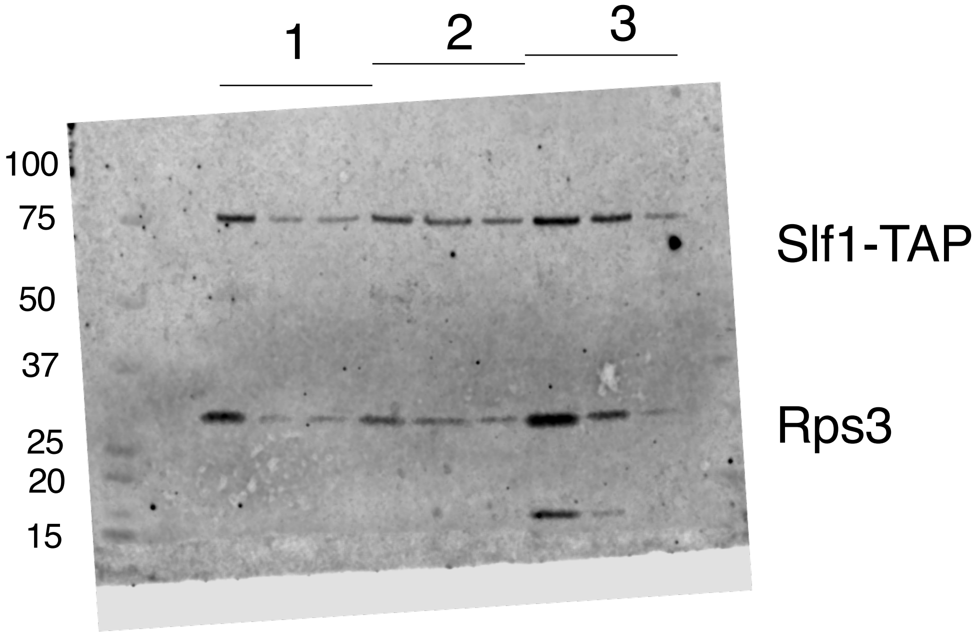

Supplementary Fig. 1B

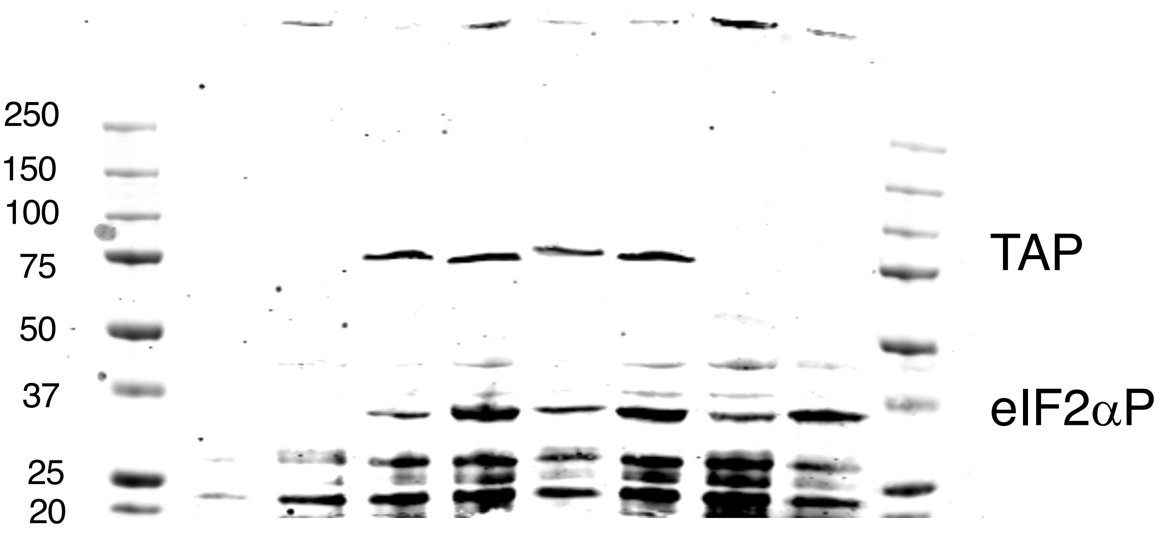

Supplementary Fig. 1C

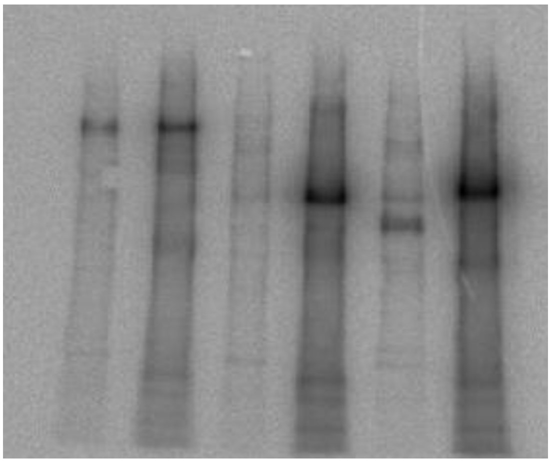

^{32}P signal

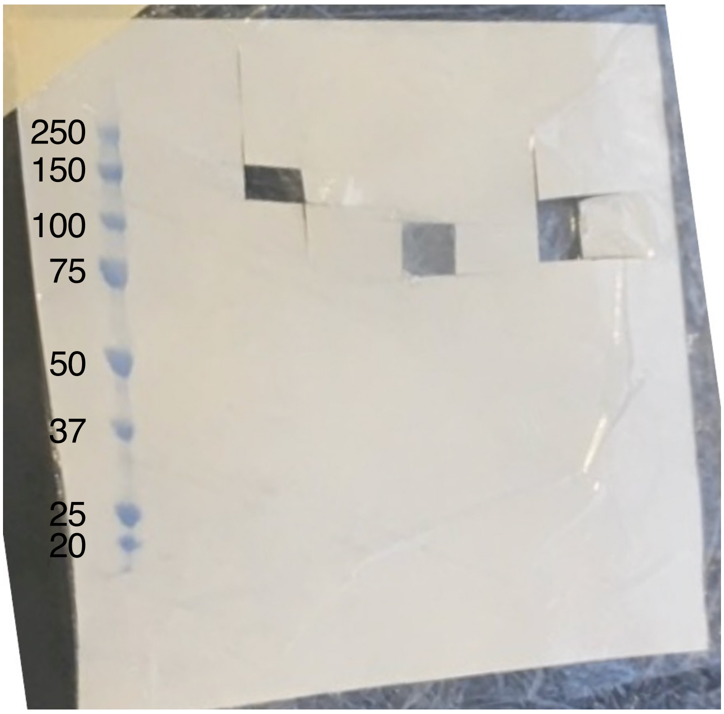

membrane following band excision

Supplementary Fig. 3C

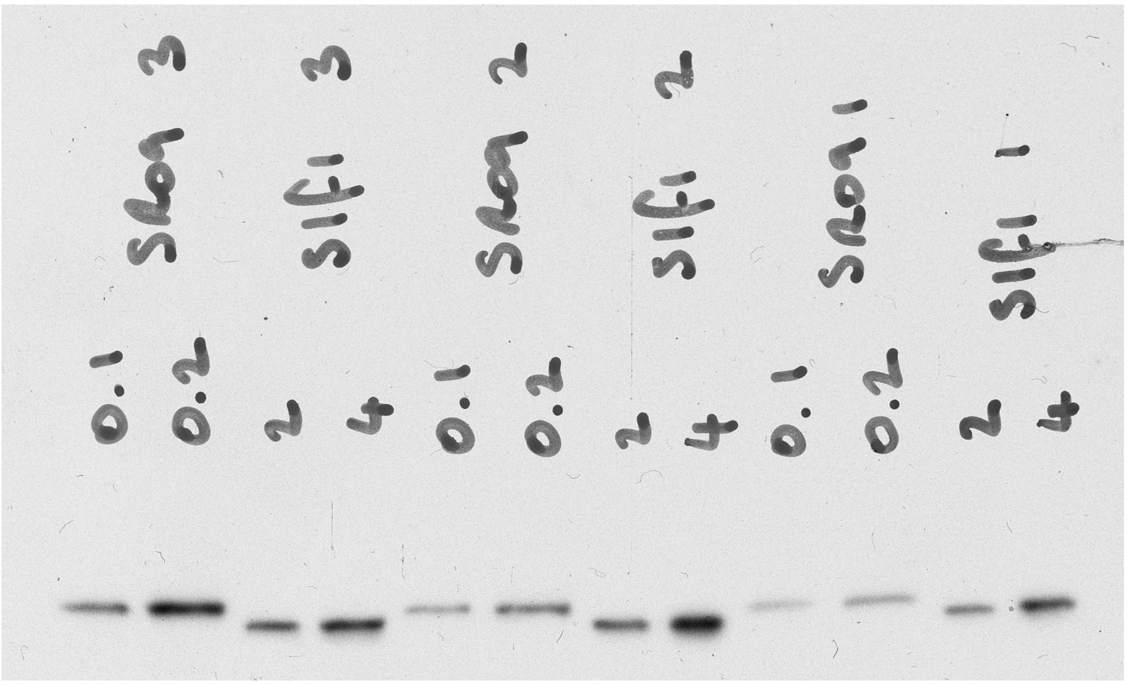

Supplementary Fig. 6D

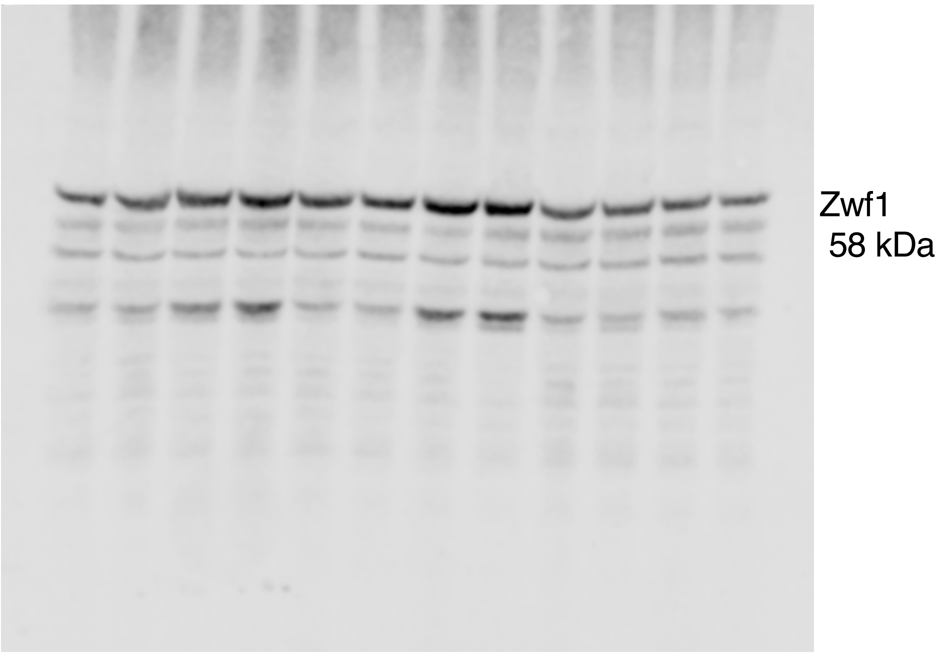

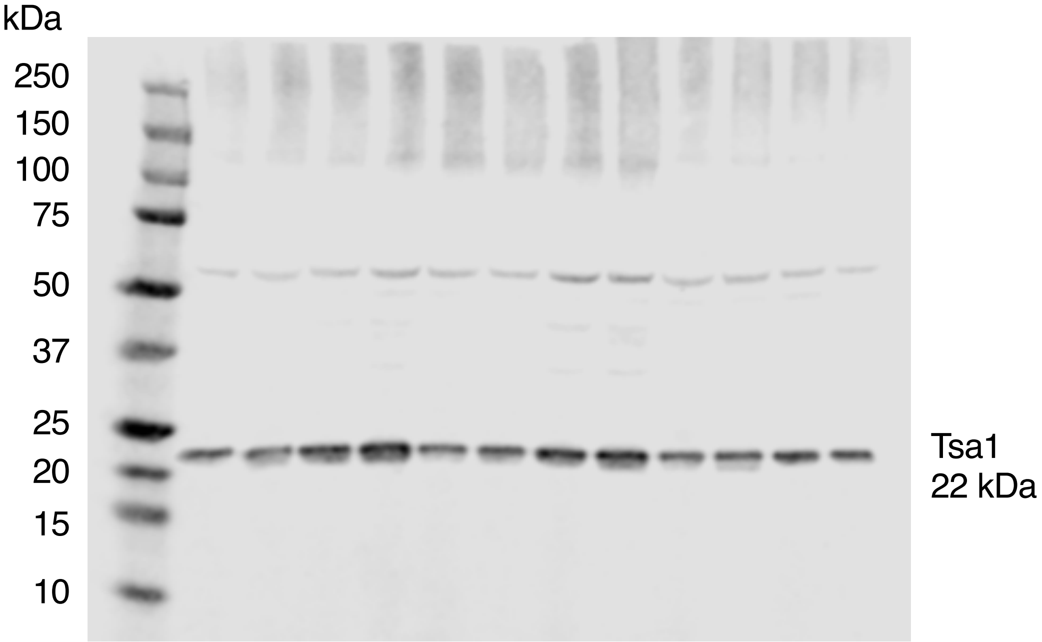

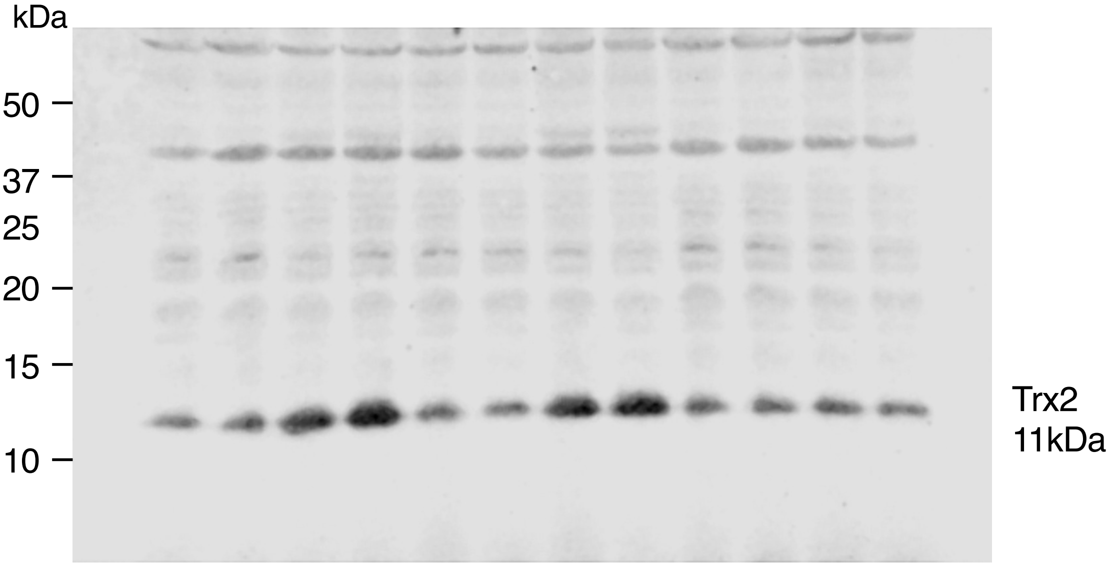

Supplementary Fig. 6D

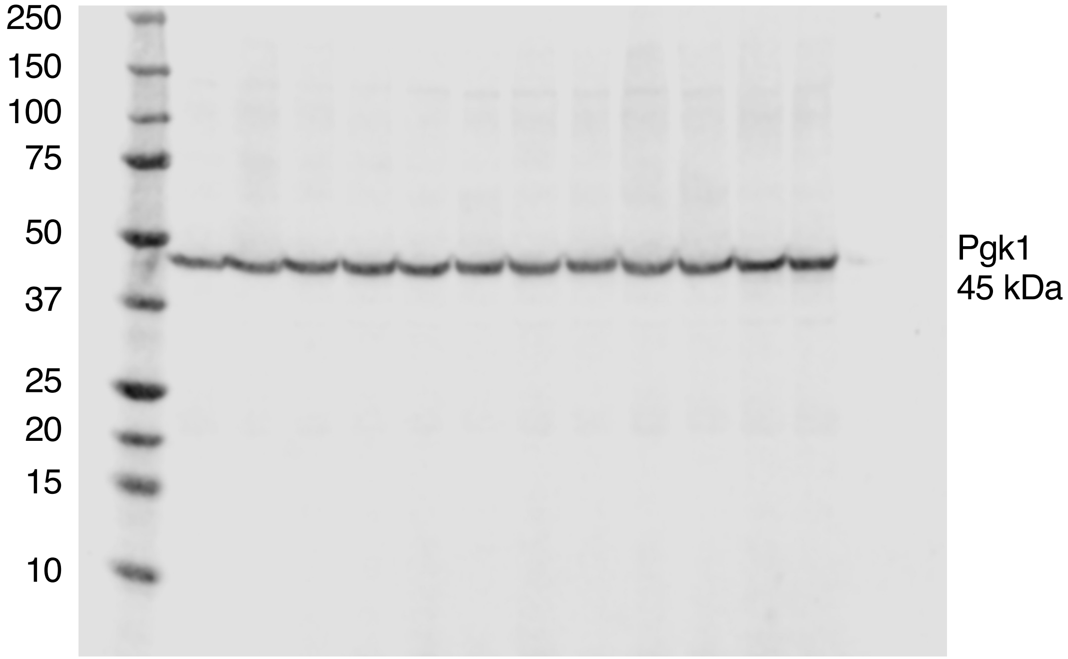

Supplementary Fig. 7C

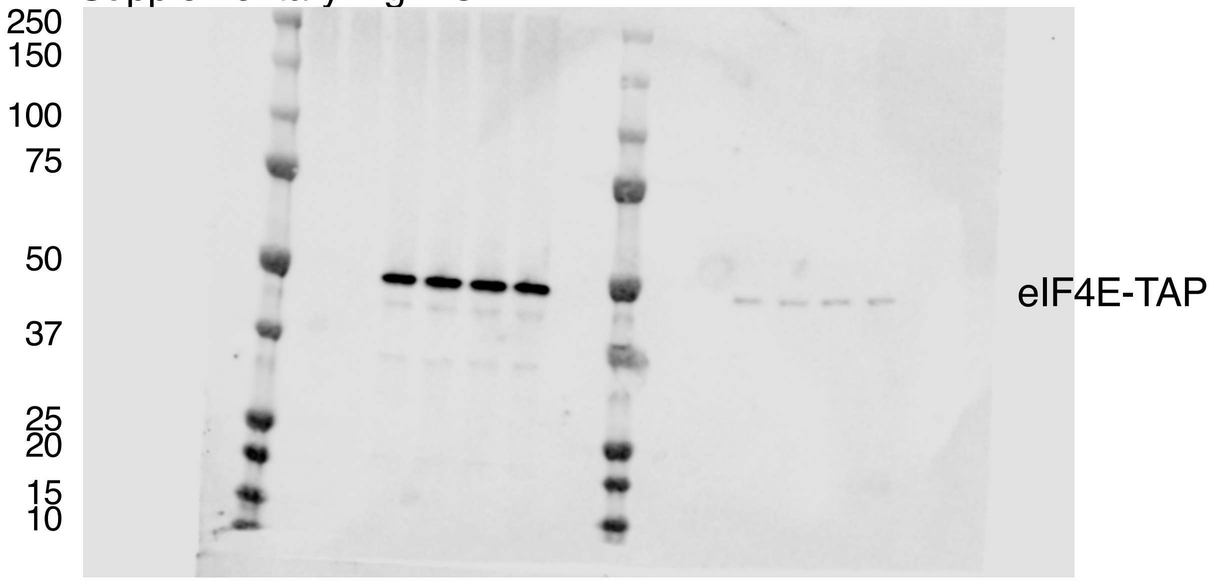

Supplementary Fig. 12A

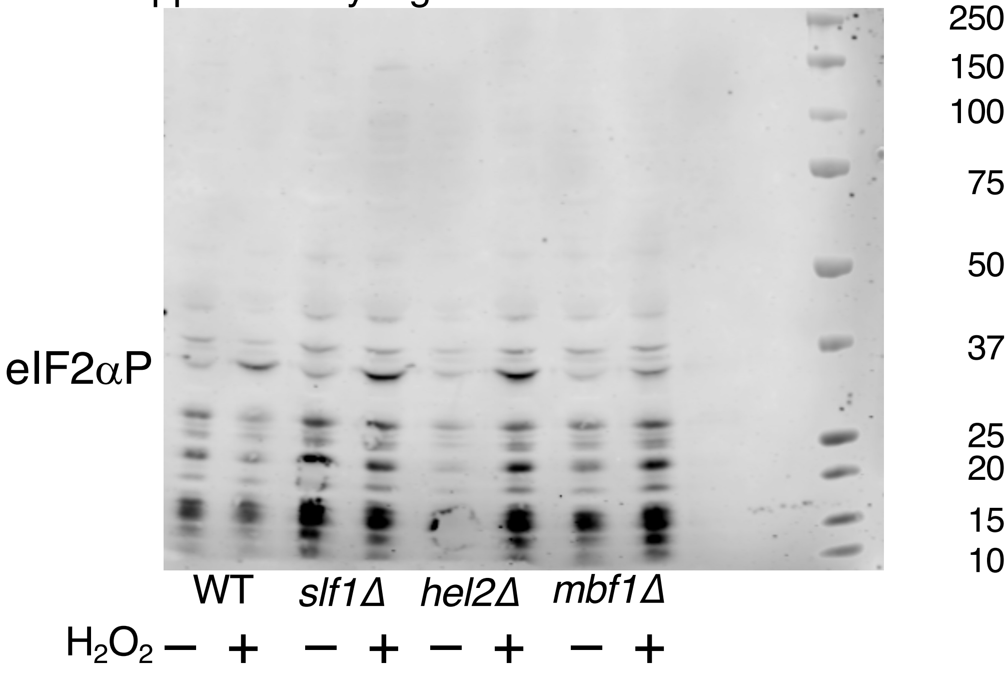

Supplementary Material References

1. Madeira, F., Pearce, M., Tivey, A.R.N., Basutkar, P., Lee, J., Edbali, O., Madhusoodanan, N., Kolesnikov, A. and Lopez, R. (2022) Search and sequence analysis tools services from EMBL-EBI in 2022. *Nucleic Acids Res.*

2. Kershaw, C.J., Costello, J., Castelli, L.M., Talavera, D., Rowe, W., Sims, P.F.G.H., Ashe, M.P., Hubbard, S.J., Pavitt, G.D. and Grant, C.M. (2015) The yeast La Related Protein Slf1p is a Key Activator of Translation During the Oxidative Stress Response *Plos Genet.*, **11**, e1004903.

3. Opitz, N., Schmitt, K., Hofer-Pretz, V., Neumann, B., Krebber, H., Braus, G.H. and Valerius, O. (2017) Capturing the Asc1p/Receptor for Activated C Kinase 1 (RACK1) Microenvironment at the Head Region of the 40S Ribosome with Quantitative BioID in Yeast. *Mol Cell Proteomics*, **16**, 2199-2218.

4. Kramer, K., Sachsenberg, T., Beckmann, B.M., Qamar, S., Boon, K.L., Hentze, M.W., Kohlbacher, O. and Urlaub, H. (2014) Photo-cross-linking and high-resolution mass spectrometry for assignment of RNA-binding sites in RNA-binding proteins. *Nat Methods*, **11**, 1064-1070.

5. dos Reis, M., Savva, R. and Wernisch, L. (2004) Solving the riddle of codon usage preferences: a test for translational selection. *Nucleic Acids Res*, **32**, 5036-5044.

6. Wu, C.C., Zinshteyn, B., Wehner, K.A. and Green, R. (2019) High-Resolution Ribosome Profiling Defines Discrete Ribosome Elongation States and Translational Regulation during Cellular Stress. *Mol Cell*, **73**, 959-970 e955.

7. Weinberg, D.E., Shah, P., Eichhorn, S.W., Hussmann, J.A., Plotkin, J.B. and Bartel, D.P. (2016) Improved Ribosome-Footprint and mRNA Measurements Provide Insights into Dynamics and Regulation of Yeast Translation. *Cell reports*, **14**, 1787-1799.

8. Costello, J., Castelli, L.M., Rowe, W., Kershaw, C.J., Talavera, D., Mohammad-Qureshi, S.S., Sims, P.F.G.H., Grant, C.M., Pavitt, G.D., Hubbard, S.J. *et al.* (2015) Global mRNA selection mechanisms for translation initiation. *Genome Biol.*, **16**, 10.

9. Meydan, S. and Guydosh, N.R. (2020) Disome and Trisome Profiling Reveal Genome-wide Targets of Ribosome Quality Control. *Mol Cell*, **79**, 588-602 e586.
